# One-pot synthesis of four-coordinate boron(III) complexes by the ligand-promoted organic group migration between boronic acids

**DOI:** 10.1038/s41598-017-00236-2

**Published:** 2017-03-21

**Authors:** Venkata S. Sadu, Hye-Rin Bin, Do-Min Lee, Kee-In Lee

**Affiliations:** 1 Major of Green Chemistry and Environmental Biotechnology, University of Science & Technology, Taejon, 305-350 South Korea; 2 Green Chemistry Division, Korea Research Institute of Chemical Technology, Taejon, 305-600 South Korea

## Abstract

Multidisciplinary applications of four-coordinate boron(III) complexes make them very attractive and challenging research field in chemistry, biology and material sciences. The dual role played by boron atom in stabilising the chelate ligand and enhancing the π-conjugation makes them very useful as luminescent materials for organic electronics and photonics, and sensing and imaging probes for biomedical purposes. The conventional methods involve the use of diarylborinic acids or anhydrides and triaryl boranes, which are made from organometallic reagents. The strong nucleophilicity of these reagents limits the peripheral modifications onto the boron cores. Here, we report a metal-free one-pot synthesis of four-coordinate organoborons using boronic acids, which represents the first instance of ligand assisted organic group migration between boronic acids. A tetrahedral boron ‘ate’ complex capable of transferring an organic group to the adjacent sp^2^ boron within a boronic anhydride intermediate is proposed and preliminary mechanistic studies by MALDI-TOF and ^11^B NMR support this proposal. The products are available from a series of *N,O*-, *N,N*- and *O,O*-bidentate ligands upon a wide array of boronic acids. We anticipate that this reaction will impact the way of producing the four-coordinate organoborons, and propel a new discovery of such materials for optoelectronic and biomedical applications.

## Introduction

Boronic acids are ubiquitous components in organic synthesis, chemical biology, and material sciences^1^. In particular, Suzuki-Miyaura cross-coupling and Chan-Lam reactions employing boronic acids or boronate esters have proven extraordinarily useful tools and are widely used in the large-scale production of pharmaceuticals and advanced materials^2, 3^. Recent advances in transition metal-catalyzed asymmetric conjugate addition and allylic arylation also emphasise the great value and versatility of boronic acids^4, 5^. The presence of the empty *p*-orbital on the boron atom renders them suitable to mediate the transfer of organic residue to a metal centre in the transmetallation step of the catalytic cross-coupling process. This unique property has been exploited with remarkable success not only in carbon-carbon and carbon-heteroatom bond formations but also in addition to unsaturated systems. Besides, the mild Lewis acidity along with their stability, low toxicity, and their ultimate degradation to environmentally friendly boric acid highlights boronic acids and their equivalents as inevitable building blocks in the transition metal-catalyzed cross-coupling reactions.

**Figure 1:**
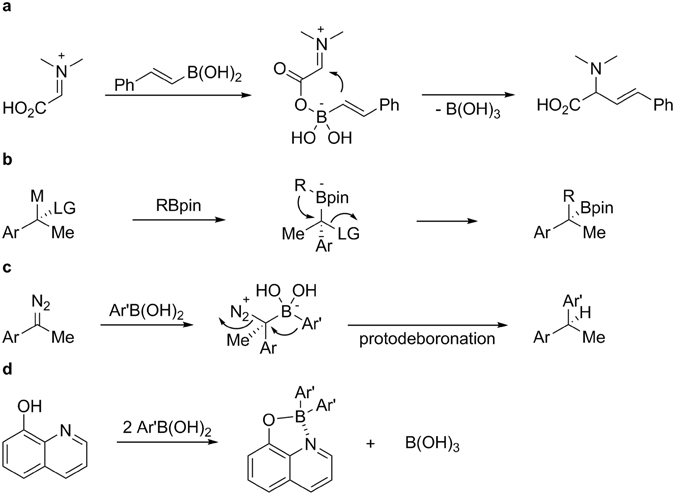
Representative examples of transition metal-free group migration from boronic acids. Group migration from boron to carbon: (**a**) Typical example of Petasis boronic acid-Mannich reaction. (**b**) Stereospecific 1,2-migration of a boronate complex to give a homologated boron intermediate bearing a quaternary stereogenic center (**c**) Reductive coupling between boronic acid and diazo compound through an α-transfer. Group migration from boron to boron: (**d**) Group migration between boronic acids in the presence of 8-hydroxyquinoline to afford a four-coordinate organoboron compound (this work). LG, leaving group, M, Li or Mg, Bpin, boronic acid pinaol ester.

Despite the amply recognised efficiency of these synthetic methods, the cost and toxicity of transition metals have urged the development of new metal-free reactions. After the pioneering work by Petasis and co-workers, multi-component reaction employing a boronic acid with an amine and an aldehyde has been extensively studied, where aryl-, alkenyl-, and alkynylboronic acids served as the nucelophilic component^6^. Most likely, the boronic acid-Mannich reaction proceeds through the initial attack of hydroxyl moiety to boronic acid leading to ‘ate’ complex, which is able to transfer the boron substituent to imine or iminium bond (Fig. 1a). There are a decent number of publications in which the boronic acids act as organic group donors under transition metal-free conditions. Along with the Petasis boronic acid-Mannich reactions, 1,2-migration of alkyl groups from boron to carbon have been developed utilising anionotropic rearrangement of tetra-substituted boron ‘ate’ complexes (Fig. 1b)^7^. Indeed, metal-free carbon-carbon bond forming reaction has been proved to be viable by the reductive coupling of tosylhydrazones (Fig. 1c) and subsequently 2,2,2-trifluorodiazoethanes with boronic acids by Barluenga and Molander, respectively^8, 9^.

Most of the rearrangements of boron ‘ate’ complexes reported in the literature involves the transfer of an organic group from the boron to the adjacent carbon. Herein we report formation of four-coordinate boron(III) complexes via an organic group migration between boronic acids in the presence of a bidentate ligand under the basic conditions (Fig. 1d). To the best of our knowledge, there is no report on the synthetic utilization of the group transfer from one to another between boronic acids.

**Figure 2:**
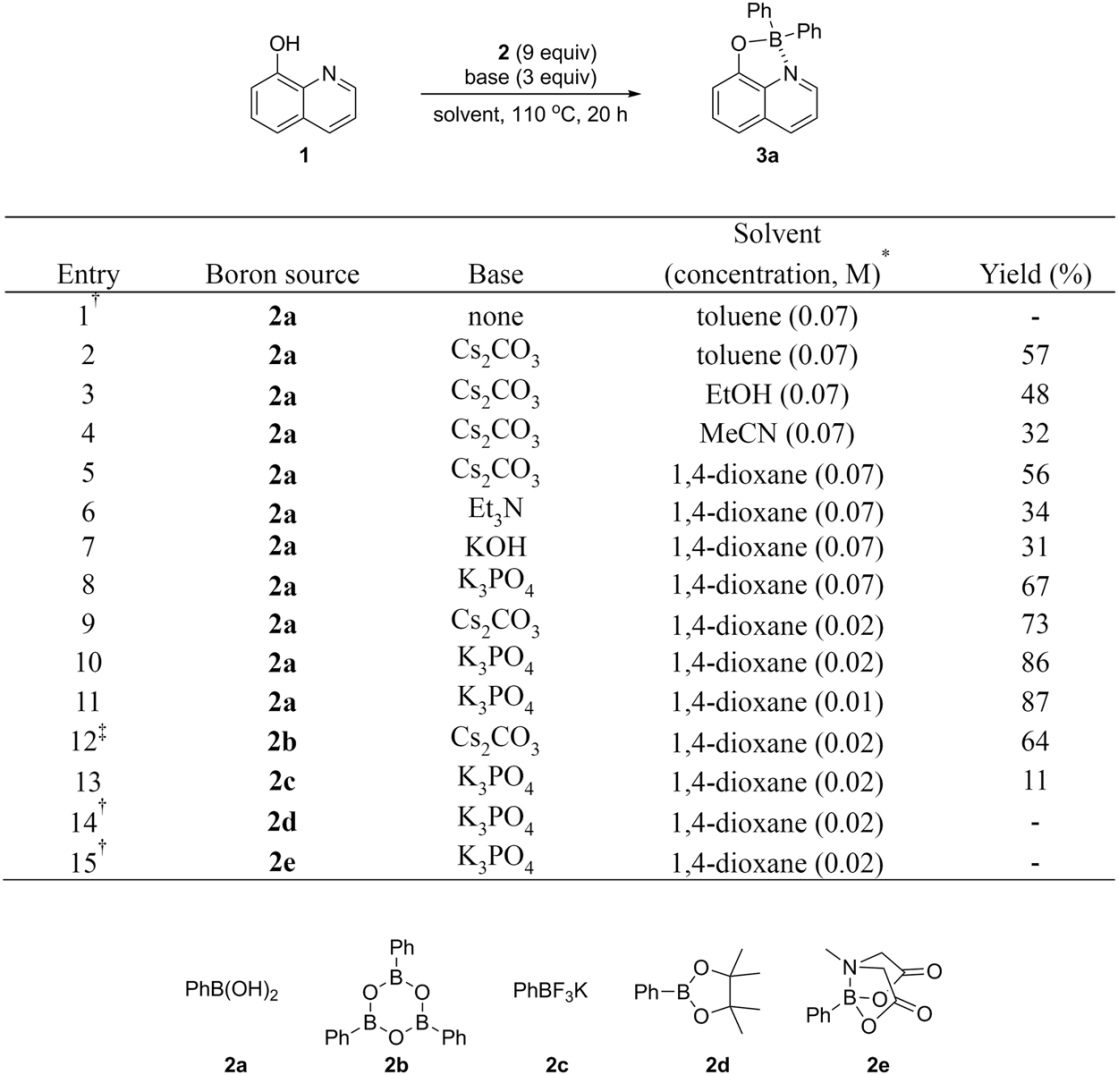
Optimization of reaction conditions. Conditions: **1** (1.0 mmol), **2** (9.0 equiv), base (3.0 equiv) in indicated solvent at 110 °C with stirring for 20 hours, unless otherwise stated. ^*^Molarity based on **1** used. ^†^No reaction observed. ^‡^3 Equiv of **2b** used.

Previously we have observed the formation of a diphenylborinate complex in the palladium-catalyzed cross-coupling reaction between 1-(2-pyridinyl)-5-trifloyloxy-1*H*-pyrazole and phenylboronic acid^10^. This was surprisingly answered that the Suzuki conditions have nothing to do with the formation of such four-coordinate boron. Encouraged by this unprecedented result, we initiated a research programme with the aim of exploring new applications of boronic acids. Four-coordinate organoboron compounds by locking with rigid π-conjugated structures have an intense luminescence and high carrier mobility. Since the boron as a core atom affects the electronic property of the molecular junction by enhancing the π-conjugation and stabilizing the anionic ligand. This enables them to be applied in optoelectronic applications including OLEDs and photovoltaic devices^11, 12^ in addition to bio-imaging and sensory materials^13–15^. However, the synthesis of four-coordinate organoboron dyes is largely dependent on the availability of boron species such as diarylborinic acids, diarylborinic anhydrides, and triarylboranes, which are currently made of pre-formed organometallic reagents^16^. The use of such reagents hampers the functional group tolerance, thus the synthesis is extremely limited in peripheral modifications onto boron cores. Keeping the variety of applications of aluminum and boron hydroxyquinolate complexes^17^ in mind we selected 8-hydroxyquinoline **1** as a model compound (Fig. 2). An initial reaction confirmed that the use of a base is essential (entry 1). The reactions worked in common solvents tested and with a variety of bases including organic and inorganic bases (entries 2–8). The yield was drastically improved by dilution (entries 9–11). From vast experimental entries, optimum conditions for the diarylborination were identified as the use of 9 equivalent of phenylboronic acid **2a** and 3 equivalents of K_3_PO_4_ under refluxing conditions in 1,4-dioxane (see Supplementary Information). While the examination of boronic acid equivalents, boroxine **2b** and potassium trifluoroborate **2c** showed low-lying yield even under the same conditions (entries 12 and 13), interestingly, pinacol and MIDA boronates (**2d** and **2e**) did not react at all (entries 14 and 15). This result is agreed well with the observation on the hydrolysis of boronic acid congeners under mild basic conditions to regenerate the corresponding boronic acid^18^.

**Figure 3:**
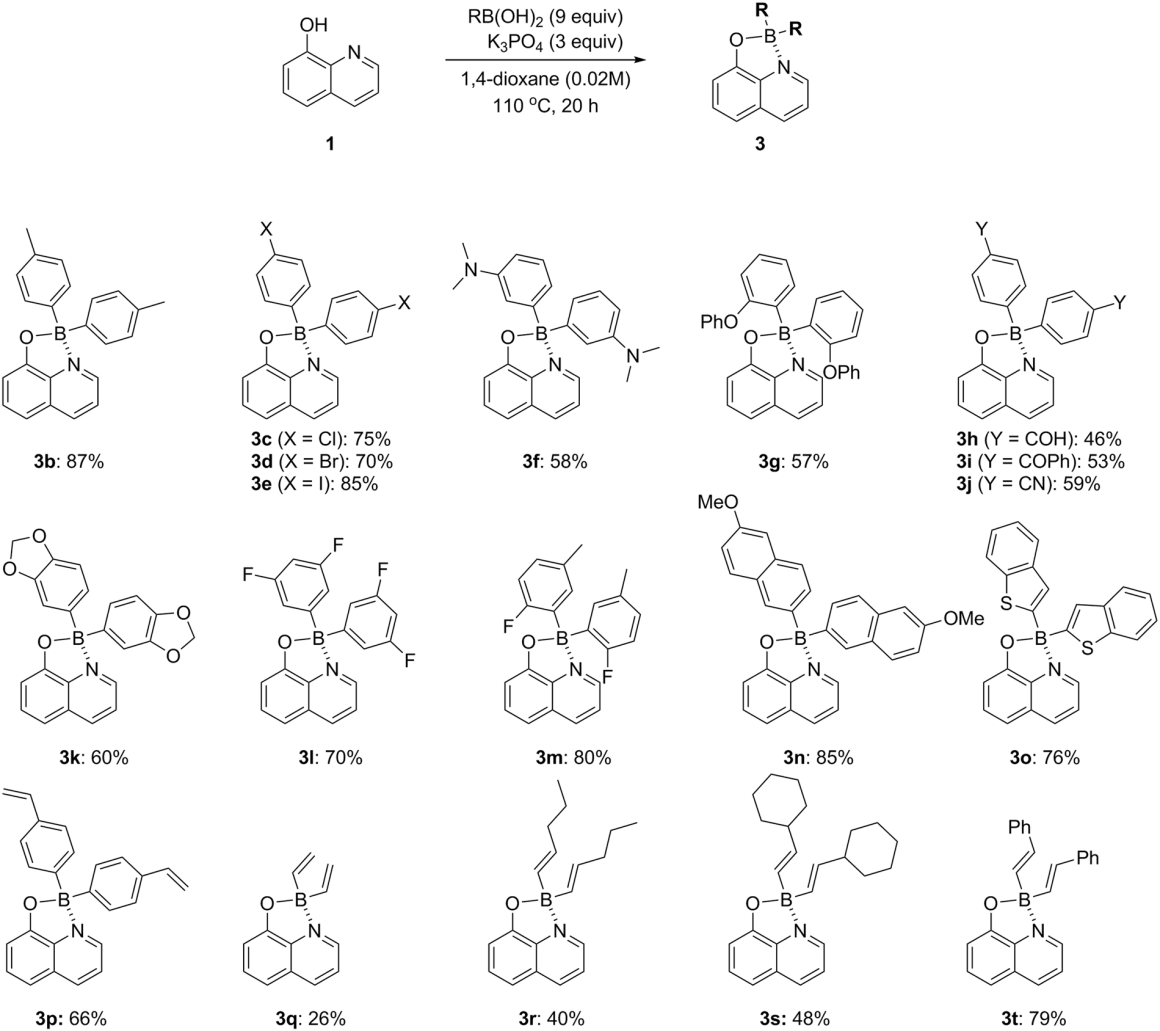
Organic group migration between boronic acids in the presence of 8-hydroxyquinoline. Conditions: **1** (1.0 mmol), boronic acid (9.0 equiv), K_3_PO_4_ (3.0 equiv) in 1,4-dioxane (0.02 M) at 110 °C with stirring for 20 hours.

The scope of the coupling partners was investigated using electronically and sterically devised aryl and alkenylboronic acids, as illustrated in Fig. 3. Substituted arylboronic acids containing methyl, dimethyamino, phenoxy, and halogen groups were compatible regardless of the substitution position (**3b**-**3g**). The halides can serve as further functional handles^19^. The Grignard reagent-susceptible groups are also tolerated (**3h**-**3j**). Disubstituted aryl and 2-naphthylboronic acids also worked well (**3k**-**3n**). However, 2,6-disubstituted boronic acids such as mesitylboronic and pentafluorophenylboronic acids did not work. The reactions show a tendency that boronic acids deactivated by electron-withdrawing groups give much higher yields. The success of 2-benzothienyl and 4-vinylphenylboronic acids demonstrates the effectiveness of this process (**3o** and **3p**)^20, 21^. We also examined the use of alkenylboronic acids with this procedure and found that it is suitable for one-pot, multi-component synthesis of the organoborons, whereas styrylboronic acid exhibits much better yield than non-stabilized alkenyl species (**3q**-**3t**). More interestingly, vinylboronic acid which has very limited shelf life also tolerated the conditions. There are no rearranged products during the group migration from boron to boron and the study for single crystals of **3f, 3h** and **3o**, revealing pseudo-tetrahedral geometry around the boron centre with the *N*,*O*-bidentate ligand, confirms this observation (see Supplementary Information). It is remarkable that simultaneous three σ-bonds and one dative bond formation could be possible in a single operation. Meanwhile, conventional synthesis heavily depends on the use of diarylborinic acids or triarylboranes. But these protocols suffer from the disadvantages associated with poor accessibility and stability. Moreover, the preparation of these boron reagents requires the use of highly reactive organolithium or Grignard reagents. This hampers the functional group compatibility thereby reducing the scope for the functionalization of peripheral groups attached to the boron atom. In view of these limitations, our finding will be a great advantage to fulfil this goal.

**Figure 4:**
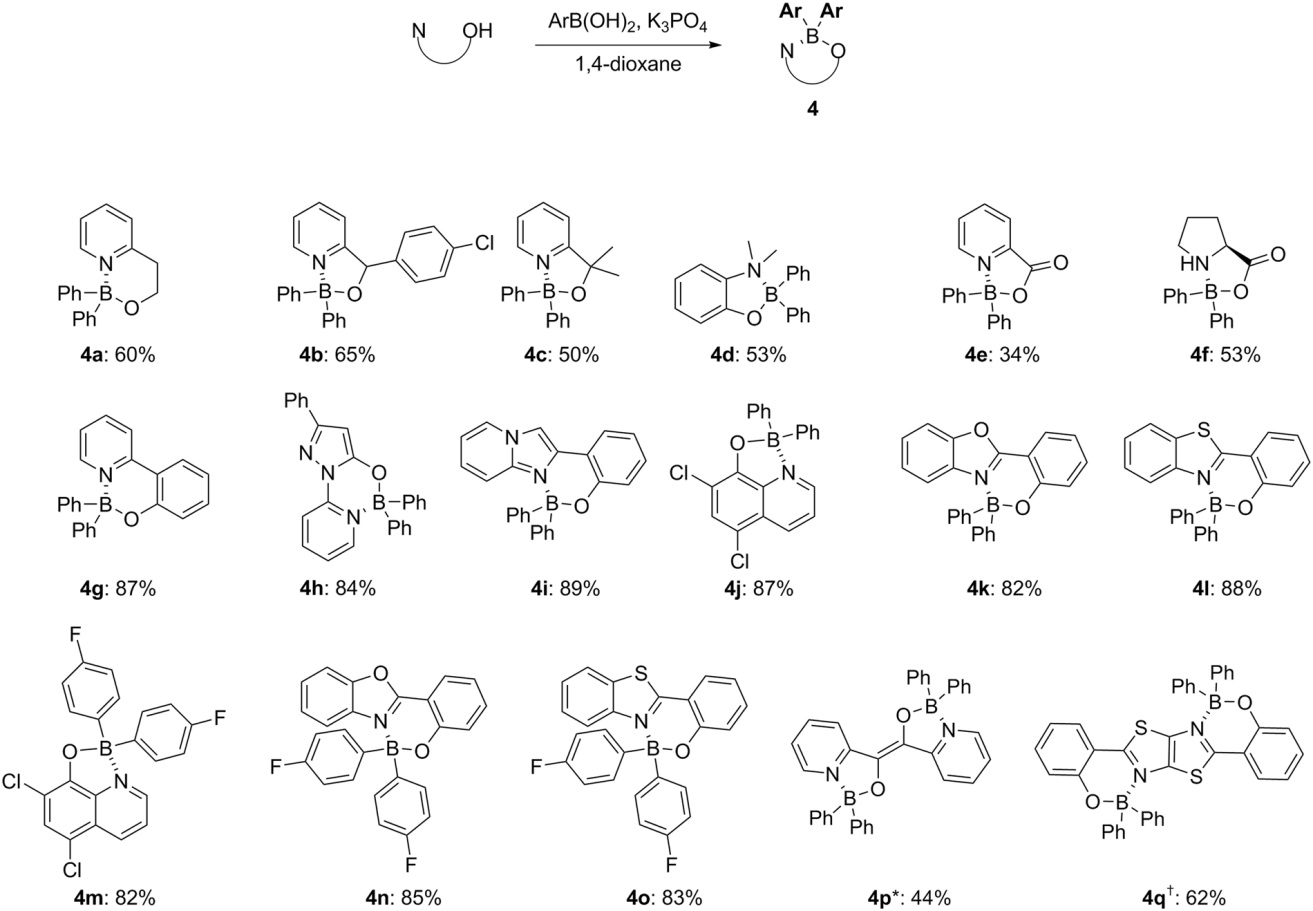
One-pot synthesis of organoborons from various *N,O*-bidentate ligands. Conditions: *N,O*-ligand (1.0 mmol), boronic acid (9.0 equiv), K_3_PO_4_ (3.0 equiv) in 1,4-dioxane (0.02 M) at 110 °C with stirring for 20 hours, unless otherwise indicated. *6 Equiv of K_3_PO_4_ and 18 equiv of phenylboronic acid were used in 1,4-dioxane (0.01 M). ^†^6 Equiv of K_3_PO_4_ and 18 equiv of phenylboronic acid were used in 1,4-dioxane (0.02 M).

In order to prove the feasibility that allows rapid access to important structural motifs, we performed the base-promoted diarylborination with various *N*,*O*-bidentate ligands (Fig. 4). Substrates ranging from primary to tertiary alcohols (**4a**-**4c**) and phenol (**4d**) worked well. Picolinic acid (**4e**) and proline (**4f**) also tolerated. All phenolic substrates showing luminescent properties gave good yields; pyridylphenol (**4g**: 87%), pyridylpyrazole (**4h**: 84%), imidazopyridine (**4i**: 89%), quinoline (**4j** and **4m**: 87% and 82%), benzoxazole (**4k** and **4n**: 82% and 85%), and benzothiazole (**4l** and **4o**: 88% and 83%). Bis-bidentate ligands (**4p** and **4q**: 44% and 62%) worked in a parallel manner, but with moderate yields. Regarding the amine part of the molecules, secondary and tertiary amines worked well, while pyridine-containing substrates exhibited the best performance. On the other hand, primary amines like ethanolamine failed. We thus revealed that one-pot synthesis of four-coordinate organoboron compounds can be extended to a wide range of *N,O*-bidentate ligands, preferentially producing five- and six-membered chelate rings. However, an attempted formation of the four-membered chelate ring from 2-hydroxypyridine was not observed. The method is viable to aryl, heteroaryl, and even alkenylboronic acids with an electronically and sterically devised fashion, thus could be used to generate a number of multi-functionalized organoboron compounds as optoelectronic and photovoltaic materials with high values.

**Figure 5:**
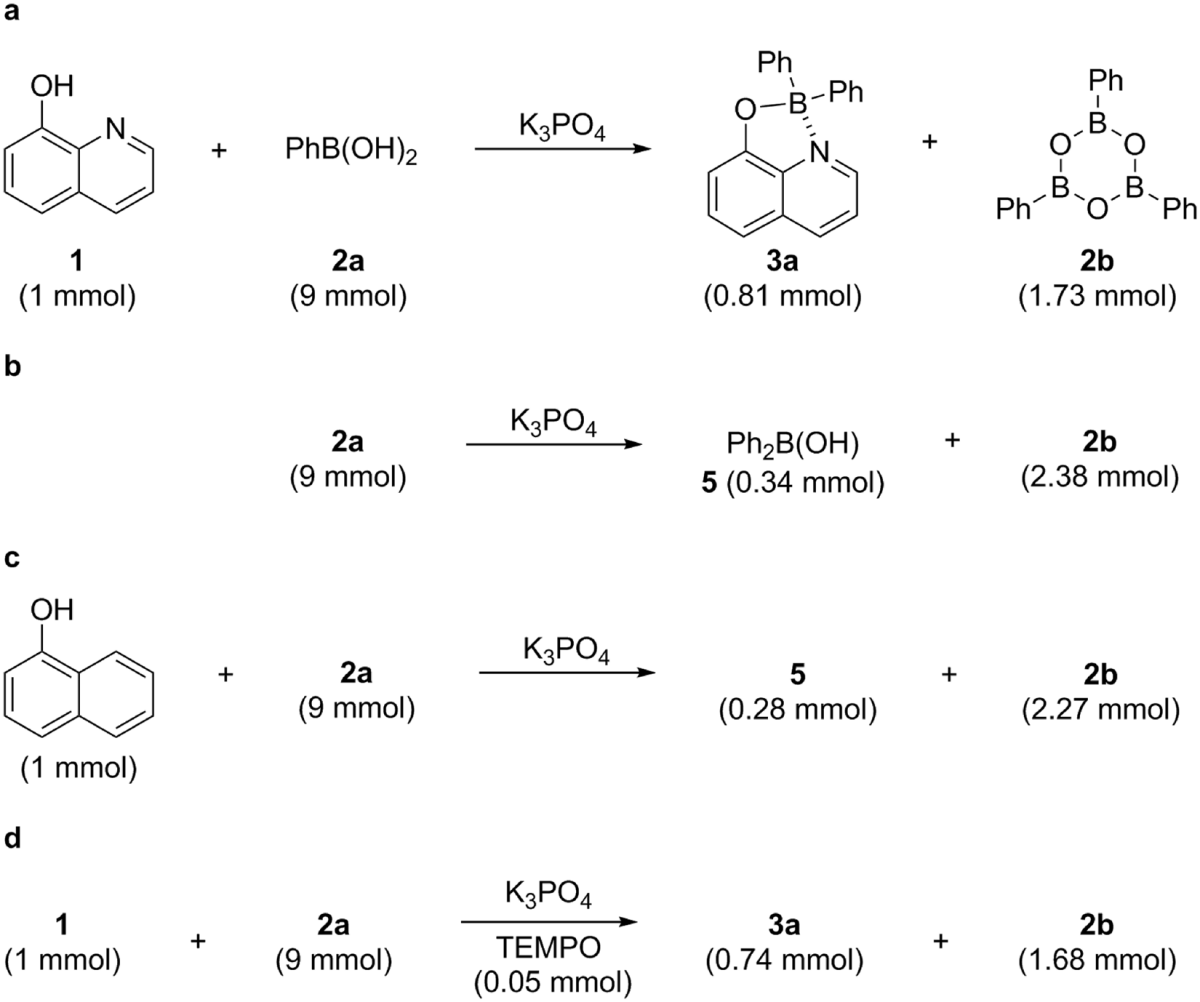
Control experiments. All reactions were performed with K_3_PO_4_ (3.0 mmol) in 1,4-dioxane (0.02 M) at 110 °C for 20 h. (**a**) The reaction suggests that product formation is more likely due to the disproportionation of phenylboronic acids facilitated by the presence of the chelate ligand under basic conditions. (**b**) This reaction demonstrates that base-induced disproportionation of boronic acids can also occur even without chelate ligand, but in lower grade. (**c**) The reaction indicates an initial formation of four-coordinated boron species assisted by a B → N dative bond is crucial for product formation. (**d**) Addition of the radical scavenger TEMPO proved to show a negligible impact on the reaction indicating that the reaction presumably proceeds by the ionic pathway.

In order to understand the unusual behaviour of boron to boron migration between boronic acids, several control experiments were investigated into the factors contributing to this unusual observation. As observed previously, the reaction of 8-hydroxyquinoline **1** with 9 equivalent of phenylboronic acid **2a** gave 0.81 mmol of **3a**, along with 1.73 mmol of triphenylboroxine **2b** (Fig. 5a). Surprisingly enough, when 9 mmol of **2a** and 3 mmol of K_3_PO_4_ were simply heated without ligand, we were able to isolate 0.34 mmol of diphenylborinic acid **5** (Fig. 5b). However, we could not observe the formation of **5** without the base. When we used 1-naphthol instead of 8-hydroxyquinoline, we realized that a chelation effect is viable in this transformation (Fig. 5c). These results confirm the role of the ligand in this transformation. In every case, excess boronic acid was recovered as its trimeric anhydride **2b** after the reaction. Although a number of possible explanations could be advanced for the present observation, these results suggest that current transformation is more likely due to the disproportionation of boronic acid facilitated by the presence of chelate ligand under basic conditions. We reasoned that an initial formation of four-coordinated boron species assisted by a B → N dative bond such as intermediate **I** (vide infra) seems to be vital. As previously observed, there was no reaction when pinacol or MIDA boronates were employed as boronic acid equivalents (entries 14–15, Fig. 2). Considering thermodynamics of boronic acids, this indicates that dimeric anhydride or oligomeric aggregate might be involved in this transformation^22–24^. Furthermore, the addition of TEMPO revealed no significant difference and this reinforced an anionic pathway to be more convincing (Fig. 5d).

**Figure 6:**
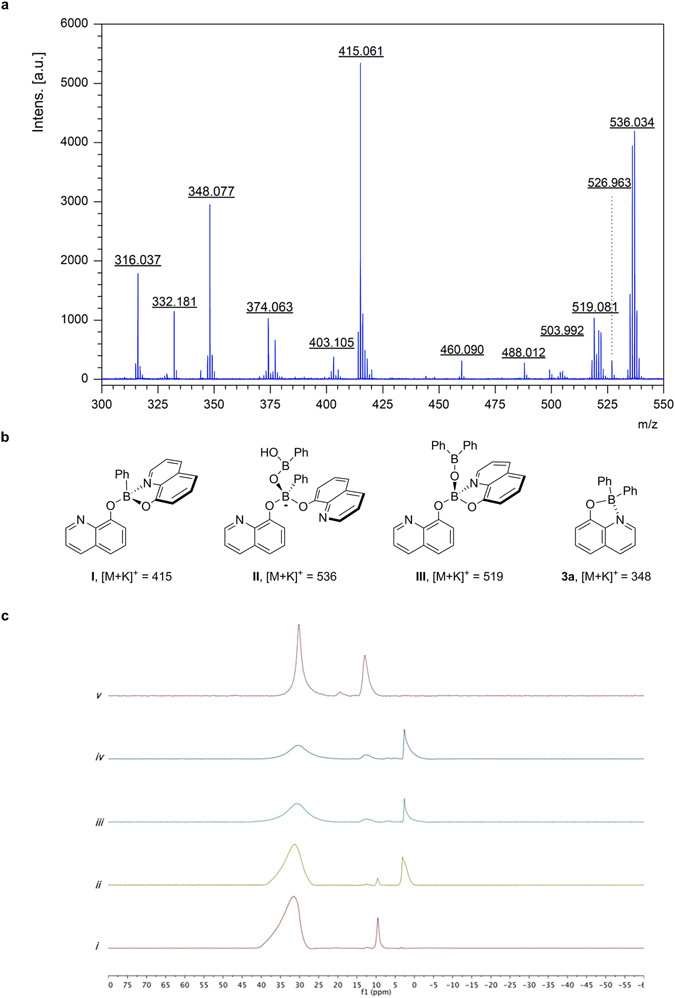
Mechanistic considerations. (**a**) MALDI-TOF mass spectrum obtained from the reaction mixture with base after 6 h. (**b**) Assigned reaction intermediates based on their mass spectral molecular weights. (**c**) ^11^B NMR spectra of the reaction mixture taken at different reaction times: without base after 16 h (***i***); and with base after 5 min (***ii***), 3 h (***iii***), 6 h (***iv***) and 9 h (***v***), respectively.

We envisioned finding some of boron intermediates from MALDI-TOF and ^11^B NMR experiments, as we believed it could provide mechanistic insight into the boron-to-boron organic group migration. An aliquot from the reaction mixtures was taken out for analysis in a time-dependent manner. Regardless of the azeotropic removal of water or not, the reaction of 8-hydroxyquinoline and PhB(OH)_2_ in toluene showed the formation of di(quinolin-8-yl) boronate and that was detected at *m/z* 377 [M + H]^+^ (see Supplementary Information). The formation of the intermediate **I** is more feasible, similar to the formation of cyclic boronates from diols and polyols^22–24^. ^11^B NMR in benzene-*d*_*6*_ showed two peaks at 9.5 and 31.5 ppm, respectively, which correspond to sp^3^-hybridized boron of **I** and sp^2^ boron of **2b** (Fig. 6c–i). However, **I** could not be isolable. After the addition of K_3_PO_4_, MALDI-TOF spectrum showed *m/z* = 415 [376 + K]^+^, 536 [497 + K]^+^, 519 [480 + K]^+^ and 348 [309 + K]^+^ (Fig. 6a). Based on their mass spectral molecular weights, we could assign possible boron intermediates corresponding to **I**, **II**, **III** and the product **3a**, respectively (Fig. 6b). This observation was further substantiated by the ^11^B NMR experiments with a time interval, which distinctively revealing a new signal centered at 2.6 ppm which correspond to sp^3^ boron of an anionic complex such as **II** (Fig. 6c). The chemical shift of sp^3^ boron of **II** is consistent with the literature values reported for the tetra-coordinated boron ‘ate’ complexes^25, 26^, however, the sp^2^ boron was not intractable since the sp^2^ boron atoms of phenylboronic acid and **2b** are essentially indistinguishable in ^11^B NMR. Upon the gradual consumption of **II**, the two peaks at 12.9 and 19.4 appeared, corresponding to the product **3a** and boric acid respectively.

Even with the experimental proof that the proposed intermediates do exist in the reaction mixtures, it's now a question of how to make organic group transfer from boron to boron and where it begins. From the spectroscopic data, we strongly believe the formation of the anionic boron complex is essential at the initial stage. The key process absolutely depends on the existence of the base which activates **I** to form the ‘ate’ complex **II** rapidly. The formation of the product **3a** was observed upon the gradual consumption of **II**. This indicates that the reaction substantially proceeds through the intermediacy of the boron ‘ate’ complex **II**, which should be more nucleophilic. The ‘ate’ complex **II** is believed to be able to transfer the organic group from the anionic boron centre onto a nearby electron-deficient sp^2^ boron through a boronic anhydride assembly. Up to date, tetrahedral boron ‘ate’ complexes are known to transfer hydride or organic groups to the electron deficient carbon atoms, found simply from borohydrides as reducing agents^27^ to Petasis boronic acid-Mannich reactions^6^. This migration event of **II** presumably ended up with the formation of trialkoxyboronate species. The observation of the borate intermediate **III** in the mass spectrum may partially answer the above question. After the event, **III** tends to rapidly hydrolyze to boric acid and borinic acid **5**, which readily couples with 8-hydroxyquinoline **1** to generate the product **3a**. Though the preliminary monitoring of the reaction supports our proposal that the boron-to-boron organic group migration occurs through the boron ‘ate’ complex pathway, however, there still remains a need for more detailed mechanistic studies.

**Figure 7:**
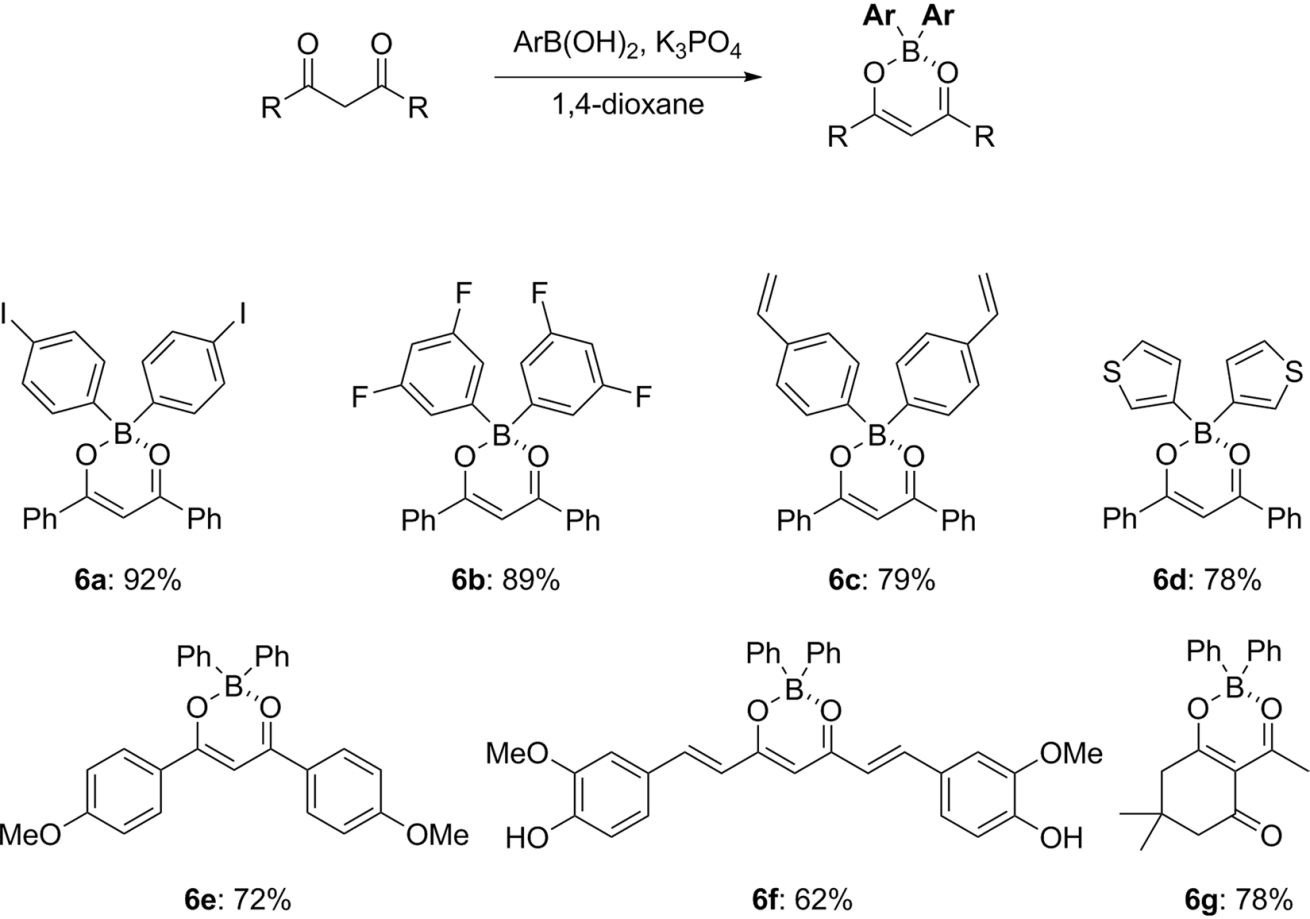
Reaction scope for organoborons from 1,3-diketones. Conditions: 1,3-diketone (1.0 mmol), boronic acid (9.0 equiv), K_3_PO_4_ (3.0 equiv) in 1,4-dioxane (0.02 M) at 110 °C with stirring for 20 hours.

**Figure 8:**
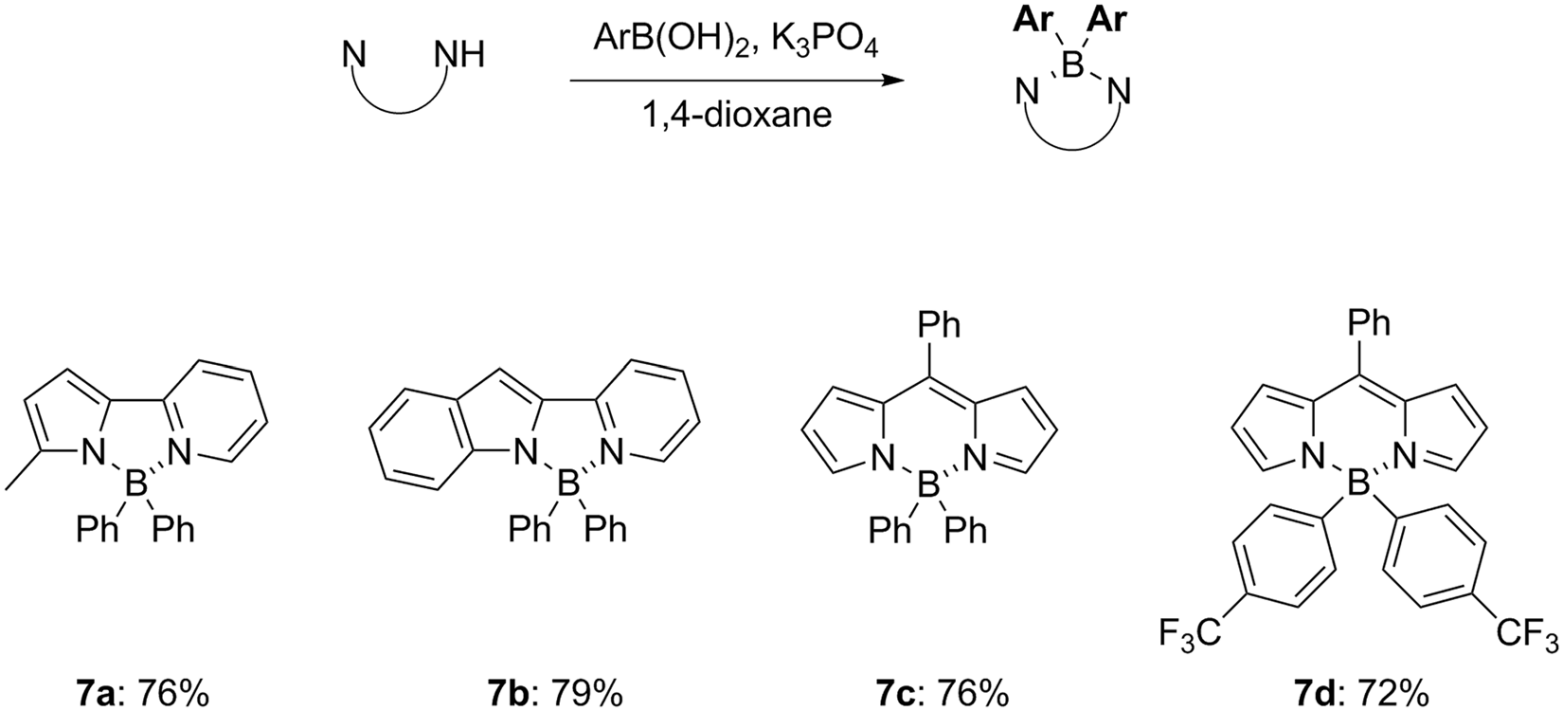
Reaction scope for organoborons from *N,N*-bidentate ligands. Conditions: *N,N*-ligand (1.0 mmol), boronic acid (9.0 equiv), K_3_PO_4_ (3.0 equiv) in 1,4-dioxane (0.02 M) at 110 °C with stirring for 20 hours.

To illuminate the synthetic utility of this process, β-diketonates and *N,N*-bidentate ligands were investigated and the results are illustrated in Figs 7 and 8, respectively. Diketone-based organoboron compounds are highly utilised as imaging agents for early detection of various human diseases^13–15^. 1,3-Diaryl-1,3-propandione substrates could be easily transformed into the corresponding diarylborinates with the representative examples of boronic acids in good yield (**6a**-**6e**). Moreover, this protocol was successfully applied to curcumin (**6f**) and also a cyclic diketone (**6g**). We next searched for the extended scope to dipyyromethene derivatives, in which boron dipyyromethene (BODIPY) dyes and their derivatives are an area of intense interest in terms of high quantum yields and molecular extinction coefficients for strong fluorescence^28, 29^. Examination also proved that it could be applicable to 2-pyrrolylpyridine and 2-pyridylindole (**7a** and **7b**) as well as BODIPY derivatives (**7c**-**d**) resulting in good yields.

In summary, we observed the first organic group migration from boron to boron between boronic acids. This migration provides an unprecedented approach to four-coordinate organoboron compounds simply employing boronic acids in the presence of bidentate ligands under basic conditions. The results suggest that the process possibly depends on the ligand-promoted disproportionation of boronic acids by the action of the base. Mechanistic studies indicate that the formation of diaryl boronate I as the key intermediate assisted by a B → N dative bond is essential. A proposed ‘ate’ complex II is believed to be able to transfer the organic group onto a nearby electron-deficient sp^2^ boron in an intramolecular fashion, resulting in the formation of borinate species. The reaction uses readily available starting materials, tolerates a variety of functional groups and operates under convenient conditions. Indeed the products are available from a series of *N,O*-, *N,N*- and *O,O*-bidentate ligands upon assembly with ranging from aryl, heteroaryl and even alkenylboronic acids. Moreover, this approach allows the introduction of functional handles such as halides, amines and carbonyl groups that can be utilised for further elaborations. Current approaches for the synthesis of four-coordinate organoborons are extremely limited particularly in peripheral modifications onto boron cores. In this respect, we firmly believe that this synthetic route can create more value for such luminescent materials due to their potential applications in material and biomedical sciences. The detailed experiments to obtain a deeper understanding of its mechanism and further applications are currently underway.

## Methods

### General

All solvents and reagents were purchased from commercial sources and used as received without further purification, unless otherwise stated. Potassium phosphate was crushed in mortar and dried at 70 °C in oven overnight and used. Reactions were monitored by thin layer chromatography carried out on S-2 0.25 mm E. Merck silica gel plates (60F-254) using UV light as the visualizing agent and an acidic mixture of anisaldehyde or a ninhydrin solution in ethanol and heat as developing agents. E. Merck silica gel (60, particle size 0.040–0.063 mm) was used for flash column chromatography. All yields were calculated from isolated products.

Melting points were recorded on Electrothermal IA9200 apparatus and are uncorrected. All NMR spectra were recorded on Bruker AV-500 instrument. ^1^H and ^13^C NMR spectra were referenced internally to the residual undeuterated chloroform (δ_H_ = 7.26 ppm and δ_C_ = 77.0 ppm). ^11^B NMR spectra were referenced externally to BF_3_·OEt_2_. The ^11^B NMR experiments were done with quartz NMR tubes (Wilmad). The NMR data were analyzed using MNova 10.0 processing software (Mestrelab Research). The following abbreviations are used to designate multiplicities: s = singlet, d = doublet, t = triplet, q = quartet, quint = quintet, m = multiplet, br s = broad singlet. Chemical shifts are reported in ppm and coupling constants are in Hertz (Hz). Infrared spectra were recorded neat on a Bruker Alpha-T FT-IR spectrometer equipped with a universal ATR sampling accessory and the wavenumber (cm^−1^) at maximum absorption of a peak is reported as ν_max_. High resolution mass spectra using Electronic Ionization (HRMS-EI) were recorded on Joel JMS-700 mass spectrometer. MALDI-TOF mass spectra were recorded on Bruker Autoflex Speed using *trans*-2-[3-(4-tert-Butylphenyl)-2-methyl-2-propenylidene]malononitrile (DCTB) under positive reflector mode. The data for X-ray structure determination were collected on Bruker SMART Apex II X-ray diffractometer equipped with graphite-monochromated MoKα radiation (λ = 0.71073 Å).

### Synthesis of Diphenyl borinic acid 8-hydroxyquinoline ester (3a)

To a 100 mL round bottomed flask equipped with magnetic stirring bar and reflux condenser, were added sequentially 8-hydroxyquinoline (145.2 mg, 1.0 mmol), PhB(OH)_2_ (1.10 g, 9.0 mmol), K_3_PO_4_ (636.8 mg, 3.0 mmol) and 1,4-dioxane (50 mL). The mixture was refluxed for 20 h, and then the solvent was evaporated under reduced pressure. The resulting crude product was taken up in EtOAc (20 mL) and water (20 mL). The separated organic layer was successively washed with 10% aq. K_3_PO_4_ solution (3 × 10 mL) and brine (10 mL), dried over anhydrous Na_2_SO_4_, and evaporated to dryness under reduced pressure. The residue was purified by column chromatography on silica gel (EtOAc/hexanes = 1/9) to afford **3a** (266 mg, 86% yield) as yellow solid.mp 205–207 °C; ^1^H NMR (500 MHz, CDCl_3_): δ_H_ 8.58 (d, *J* = 4.89 Hz, 1 H), 8.40 (d, *J* = 8.25 Hz, 1 H), 7.68–7.60 (m, 2 H), 7.30–7.22 (m, 7 H), 7.18 (d, *J* = 7.77 Hz, 1 H) ppm; ^13^C NMR (125 MHz, CDCl_3_): δ_C_ 158.8, 139.3, 138.7, 137.6, 132.9, 132.0, 128.5, 127.6, 127.0, 122.7, 112.2, 109.7 ppm; ^11^B NMR (160 MHz, CDCl3): δ_B_ 13.2 ppm. HRMS-EI *m/z* [M]^+^ calcd for C_21_H_16_NOB, 309.1325, found 309.1323.

## Electronic supplementary material

One-pot synthesis of four-coordinate boron(III) complexes by the ligand-promoted organic group migration between boronic acids
