# Supplementary material for: One-pot synthesis of four-coordinate boron(III) complexes by the ligand-promoted organic group migration between boronic acids
Source: Sci Rep. 2017 Mar 21;7:242. doi: 10.1038/s41598-017-00236-2 (PMC5427980; doi:10.1038/s41598-017-00236-2)
Supplement: Supplementary file 1 — One-pot synthesis of four-coordinate boron(III) complexes by the ligand-promoted organic group migration between boronic acids [file 41598_2017_236_MOESM1_ESM.doc]

**SUPPLEMENTARY INFORMATION**

**One-pot synthesis of four-coordinate boron(III) complexes by the ligand-promoted organic group migration between boronic acids**

Venkata S. Sadu, Hye-Rin Bin, Do-Min Lee & Kee-In Lee^*^

Green Chemistry Division, Korea Research Institute of Chemical Technology, Taejon 305-600, South Korea

E-mail: [kilee@krict.re.kr](mailto:kilee@krict.re.kr)

**Table of Contents**

1. General Information …………………………………………………………….……2
2. Optimization of Reaction Conditions ……………………………………………..…3
3. Preparation of Four-Coordinate Organoborons from *N,O*-Ligands ……………….…5
4. 8-Hydroxyquinoline Derivatives ……………………………………………..5
5. X-Ray Crystal Structures ……………………………………………..……..13
6. Various *N,O*-type Organoborons ……………………………………..……..16
7. Mechanistic Considerations ………………………………………………..…….…22
8. Control Experiments ……………………………………………...…………22
9. MALDI-TOF and ^11^B NMR Studies ……...……………………………....…28
10. Preparation of Four-Coordinate Organoborons from *O,O*-Ligands ……….........…..34
11. Preparation of Four-Coordinate Organoborons from *N,N*-Ligands ………...………37
12. References …………………………………………………………...…….………39
13. Copies of NMR Spectra ………………………………………..………………..40

**I. General Information**

All solvents and reagents were purchased from commercial sources and used as received without further purification, unless otherwise stated. Potassium phosphate was crushed in mortar and dried at 70°C in oven overnight and used. Reactions were monitored by thin layer chromatography carried out on S-2 0.25 mm E. Merck silica gel plates (60F-254) using UV light as the visualizing agent and an acidic mixture of anisaldehyde or a ninhydrin solution in ethanol and heat as developing agents. E. Merck silica gel (60, particle size 0.040-0.063 mm) was used for flash column chromatography. All yields were calculated from isolated products.

Melting points were recorded on Electrothermal IA9200 apparatus and are uncorrected. All NMR spectra were recorded on Bruker AV-500 instrument. ^1^H and ^13^C NMR spectra were referenced internally to the residual undeuterated chloroform (δ_H_ = 7.26 ppm and δ_C_ = 77.0 ppm). ^11^B NMR spectra were referenced externally to BF_3_**^.^**OEt_2_. The ^11^B NMR experiments were done with quartz NMR tubes (Wilmad). The NMR data were analyzed using MNova 10.0 processing software (Mestrelab Research). The following abbreviations are used to designate multiplicities: s = singlet, d = doublet, t = triplet, q = quartet, quint = quintet, m = multiplet, br s = broad singlet. Chemical shifts are reported in ppm and coupling constants are in Hertz (Hz). Infrared spectra were recorded neat on a Bruker Alpha-T FT-IR spectrometer equipped with a universal ATR sampling accessory and the wavenumber (cm^-1^) at maximum absorption of a peak is reported as ν_max_. High resolution mass spectra using Electronic Ionization (HRMS-EI) were recorded on Joel JMS-700 mass spectrometer. MALDI-TOF mass spectra were recorded on Bruker Autoflex Speed using *trans*-2-[3-(4-tert-Butylphenyl)-2-methyl-2-propenylidene]malononitrile (DCTB) under positive reflector mode. The data for X-ray structure determination were collected on Bruker SMART Apex II X-ray diffractometer equipped with graphite-monochromated MoKα radiation (λ = 0.71073 Å).

**II. Optimization of Reaction Conditions**

In every case the experiments were performed on 1 mmol-scale using 8-hydroxyquinoline, and M indicates the molarity of 8-hydroxyquinoline.

Table S1. Variation in quantity of reaction components

| Entry | Solvent | Equiv of PhB(OH)_2_ | Equiv of Cs_2_CO_3_ | Concentration  (M) | Yield (%) |
| --- | --- | --- | --- | --- | --- |
| 1 | toluene | 3 | none | 0.07 | - |
| 2 | EtOH | 3 | 1 | 0.07 | 17 |
| 3 | xylenes | 4 | 3 | 0.07 | 29 |
| 4 | toluene | 4 | 3 | 0.07 | 37 |
| 5 | toluene | 9 | 1 | 0.07 | 19 |
| 6 | toluene | 9 | 3 | 0.07 | 57 |
| 7 | 1,4-dioxane | 9 | 3 | 0.07 | 56 |
| 8 | 1,4-dioxane | 11 | 3 | 0.07 | 55 |

Table S2. Effect of solvent

| Entry | Solvent | Equiv of PhB(OH)_2_ | Equiv of Cs_2_CO_3_ | Concentration  (M) | Yield (%) |
| --- | --- | --- | --- | --- | --- |
| 1 | toluene | 9 | 3 | 0.07 | 57 |
| 2 | xylenes | 9 | 3 | 0.07 | 44 |
| 3 | EtOH | 9 | 3 | 0.07 | 48 |
| 4 | 1,4-dioxane | 9 | 3 | 0.07 | 56 |
| 5 | MeCN | 9 | 3 | 0.07 | 32 |
| 6 | DMF | 9 | 3 | 0.07 | 58 |
| 7 | DMSO | 9 | 3 | 0.07 | 26 |

Table S3. Choice of base and effect of concentration

| Entry | Solvent | Equiv of PhB(OH)_2_ | Base (equiv) | Concentration  (M) | Yield (%) |
| --- | --- | --- | --- | --- | --- |
| 1 | 1,4-dioxane | 9 | Et_3_N (3) | 0.07 | 34 |
| 2 | 1,4-dioxane | 9 | *i*-Pr_2_NEt (3) | 0.07 | 20 |
| 3 | 1,4-dioxane | 9 | Cs_2_CO_3_ (3) | 0.07 | 56 |
| 4 | 1,4-dioxane | 9 | Cs_2_CO_3_ (3) | 0.02 | 73 |
| 6 | 1,4-dioxane | 9 | Cs_2_CO_3_ (3) | 0.01 | 76 |
| 7 | 1,4-dioxane | 9 | K_3_PO_4_ (3) | 0.07 | 67 |
| 8 | 1,4-dioxane | 9 | K_3_PO_4_ (3) | 0.02 | 86 |
| 9 | 1,4-dioxane | 9 | K_3_PO_4_ (3) | 0.01 | 87 |
| 10 | 1,4-dioxane | 9 | KOH (3) | 0.07 | 31 |
| 11 | 1,4-dioxane | 9 | KOH (3) | 0.02 | 77 |

Table S4. Effect of base quantity

| Entry | Solvent | Equiv of PhB(OH)_2_ | Equiv of  K_3_PO_4_ | Concentration  (M) | Yield (%) |
| --- | --- | --- | --- | --- | --- |
| 1 | 1,4-dioxane | 9 | 1 | 0.02 | 55 |
| 2 | 1,4-dioxane | 9 | 2 | 0.02 | 73 |
| 3 | 1,4-dioxane | 9 | 3 | 0.02 | 86 |
| 4 | 1,4-dioxane | 9 | 4 | 0.02 | 84 |
| 5 | 1,4-dioxane | 9 | 7 | 0.02 | 78 |

Table S5. Effect of boronic acid quantity

| Entry | Solvent | Equiv of PhB(OH)_2_ | Equiv of  K_3_PO_4_ | Concentration  (M) | Yield (%) |
| --- | --- | --- | --- | --- | --- |
| 1 | 1,4-dioxane | 2 | 3 | 0.02 | 31 |
| 2 | 1,4-dioxane | 3 | 3 | 0.02 | 43 |
| 3 | 1,4-dioxane | 5 | 3 | 0.02 | 64 |
| 4 | 1,4-dioxane | 6 | 3 | 0.02 | 70 |
| 5 | 1,4-dioxane | 9 | 3 | 0.02 | 86 |
| 6 | 1,4-dioxane | 18 | 3 | 0.02 | 71 |

**III. Preparation of Four-Coordinate Organoborons from *N,O*-Ligands**

**A. 8-Hydroxyquinoline Derivatives**

**General Procedure for the preparation of 3a**

To a 100 mL round bottomed flask equipped with magnetic stirring bar and reflux condenser, were added sequentially 8-hydroxyquinoline (145.2 mg, 1.0 mmol), PhB(OH)_2_ (1.10 g, 9.0 mmol), K_3_PO_4_ (636.8 mg, 3.0 mmol) and 1,4-dioxane (50 mL). The mixture was refluxed for 20 h, and then the solvent was evaporated under reduced pressure. The resulting crude product was taken up in EtOAc (20 mL) and water (20 mL). The separated organic layer was successively washed with 10% aq. K_3_PO_4_ solution (3 x 10 mL) and brine (10 mL), dried over anhydrous Na_2_SO_4_, and evaporated to dryness under reduced pressure. The residue was purified by column chromatography on silica gel (EtOAc/hexanes = 1/9) to afford **3a** (266 mg, 86%) as yellow solid.

**Diphenyl borinic acid 8-hydroxyquinoline ester (3a)**

mp 205-207 °C (lit.^1^ 205 °C); ^1^H NMR (500 MHz, CDCl_3_): δ_H_ 8.58 (d, *J* = 4.89 Hz, 1H), 8.40 (d, *J* = 8.25 Hz, 1H), 7.68-7.60 (m, 2H), 7.30-7.22 (m, 7H), 7.18 (d, *J* = 7.77 Hz, 1H) ppm; ^13^C NMR (125 MHz, CDCl_3_): δ_C_ 158.8, 139.3, 138.7, 137.6, 132.9, 132.0, 128.5, 127.6, 127.0, 122.7, 112.2, 109.7 ppm; ^11^B NMR (160 MHz, CDCl3): δ_B_ 13.2 ppm; HRMS-EI *m/z* [M]^+^ calcd for C_21_H_16_NOB, 309.1325, found 309.1323.

**Di-(*p*-tolyl)borinic acid 8-hydroxyquinoline ester (3b)**

293 mg, 87%; yellow solid; mp 195-196 °C; ^1^H NMR (500 MHz, CDCl_3_): δ_H_ 8.58 (d, *J* = 4.65Hz, 1H), 8.39 (d, *J* = 8.35 Hz, 1H), 7.68 (t, *J* = 8.1 Hz, 1H), 7.61 (dd, *J* = 4.95, 8.4 Hz, 1H), 7.39 (d, *J* = 7.85 Hz, 4H), 7.25 (d, *J* = 8.35 Hz, 1H), 7.19 (d, *J* = 7.55 Hz, 1H), 7.14 (d, *J* = 7.85 Hz, 4H), 2.32 (s, 6H) ppm; ^13^C NMR (125 MHz, CDCl_3_): δ_C_ 158.9, 139.2, 138.6, 137.6, 136.4, 132.9, 132.1, 128.5, 128.4, 122.7, 112.1, 109.6, 21.3 ppm; HRMS-EI *m/z* [M]^+^ calcd for C_23_H_20_NOB, 337.1638, found 337.1633.

**Di-(4-chlorophenyl)borinic acid 8-hydroxyquinoline ester (3c)**

284 mg, 75%; yellow solid; mp 191-193 °C (lit.^2^ 192-194 °C); ^1^H NMR (500 MHz, CDCl_3_): δ_H_ 8.59 (d, *J* = 4.65 Hz, 1H), 8.40-8.38 (m, 1H), 7.68 (t, *J* = 8.1 Hz, 1H), 7.61 (dd, *J* = 4.95, 8.4 Hz, 1H), 7.40 (d, *J* = 7.85 Hz, 4H), 7.25 (d, *J* = 8.35 Hz, 1H), 7.19 (d, *J* = 7.55 Hz, 1H,), 7.14 (d, *J* = 7.85 Hz, 4H) ppm; ^13^C NMR (125 MHz, CDCl_3_): δ_C_ 158.4, 139.2, 137.5, 137.0, 133.4, 133.2, 133.1, 128.5, 127.8, 122.9, 112.7, 110.0 ppm; HRMS-EI *m/z* [M]^+^ calcd for C_21_H_14_Cl_2_NOB, 377.0545, found 377.0537

**Di-(4-bromophenyl)borinic acid 8-hydroxyquinoline ester (3d)**

327 mg, 70%; yellow solid; mp 200-201 °C; ^1^H NMR (500 MHz, CDCl_3_) δ_H_ 8.51 (dd, *J* = 5.1, 1.0 Hz, 1H), 8.44 (dd, *J* = 8.4, 1.0 Hz, 1H), 7.73-7.61 (m, 2H), 7.44-7.35 (m, 4H), 7.30-7.25 (m, 5H), 7.19 (d, *J* = 7.7 Hz, 1H) ppm; ^13^C NMR (125 MHz, CDCl_3_): δ_C_ 158.4, 139.2, 139.2, 137.4, 133.7, 133.1, 130.8, 130.7, 130.6, 128.5, 122.9, 121.6, 112.7, 110.0; HRMS-EI *m/z* [M]^+^ calcd for C_21_H_14_Br_2_NOB, 464.9535, found 464.9552.

**Di-(4-iodophenyl)borinic acid 8-hydroxyquinoline ester (3e)**

477 mg, 85%; yellow solid; mp 189-192°C; ^1^H NMR (500 MHz, CDCl_3_) δ_H_ 8.50 (dd, *J* = 5.1, 1.0 Hz, 1H), 8.44 (dd, *J* = 8.4, 1.0 Hz, 1H), 7.71-7.62 (m, 2H), 7.62-7.56 (m, 4H), 7.28 (d, *J* = 8.3 Hz, 1H), 7.18 (d, *J* = 7.7 Hz, 1H), 7.16 -7.10 (m, 4H) ppm; ^13^C NMR (125 MHz, CDCl_3_): δ_C_ 158.4, 139.2, 139.1, 137.5, 136.7, 133.9, 133.1, 128.5, 122.9, 112.7, 110.0, 93.6 ppm; HRMS-EI *m/z* [M]^+^ calcd for C_21_H_14_NOBI_2_, 560.9258, found 560.9254

**Di-(3-dimethylaminophenyl)borinic acid 8-hydroxyquinoline ester (3f)**

229 mg, 58%; yellow solid; mp 149-150 °C; ^1^H NMR (500 MHz, CDCl_3_) δ_H_ 8.61 (dd, *J* = 5.0, 1.0 Hz, 1H), 8.36 (dd, *J* = 8.3, 1.0 Hz, 1H), 7.64 (t, *J* = 8.0 Hz, 1H), 7.58 (dd, *J* = 8.3, 5.0 Hz, 1H), 7.21 (d, *J* = 8.3 Hz, 1H), 7.16 (t, *J* = 7.7 Hz, 3H), 7.06-6.96 (m, 2H), 6.80 (dt, *J* = 7.2, 1.1 Hz, 2H), 6.66 (ddd, *J* = 8.2, 2.8, 1.0 Hz, 2H), 2.87 (s, 12H); ^13^C NMR (125 MHz, CDCl_3_): δ_C_ 159.0, 150.2, 139.3, 138.5, 137.7, 132.8, 128.4, 128.2, 122.6, 120.9, 117.3, 112.0, 111.9, 109.5, 41.0 ppm; ^11^B NMR (160 MHz, CDCl_3_): δ_B_ 13.1 ppm; HRMS-EI *m/z* [M]^+^ calcd for C_25_H_26_N_3_OB, 395.2169, found 395.2173.

**Di-(2-phenoxyphenyl)borinic acid 8-hydroxyquinoline ester** **(3g)**

281 mg, 57%; yellow solid; mp 174-175 °C; ^1^H NMR (500 MHz, CDCl_3_) δ_H_ 8.96 (dd, *J* = 5.2, 1.0 Hz, 1H), 8.05 (dd, *J* = 8.2, 1.0 Hz, 1H), 7.75 (dd, *J* = 7.4, 1.8 Hz, 2H), 7.48 (t, *J* = 8.0 Hz, 1H), 7.21-7.12 (m, 3H), 7.11-7.04 (m, 7H), 6.96 (d, *J* = 7.6 Hz, 1H), 6.93-6.88 (m, 2H), 6.76-6.70 (m, 2H), 6.55-6.49 (m, 4H) ppm; ^13^C NMR (125 MHz, CDCl_3_): δ_C_ 159.4, 158.7, 157.3, 141.8, 198.1, 137.9, 135.9, 131.9, 129.1, 128.5, 128.1, 123.0, 122.1, 121.9, 118.1, 117.9, 111.9, 108.9 ppm; ^11^B NMR (160 MHz, CDCl_3_): δ_B_ 12.3 ppm; HRMS-EI *m/z* [M]^+^ calcd for C_33_H_24_NO_3_B, 493.1849, found 493.1854.

**Di-(4-formylphenyl)borinic acid 8-hydroxyquinoline ester (3h)**

168 mg, 46%; yellow solid; mp 151-152 °C; ^1^H NMR (500 MHz, CDCl_3_) δ_H_ 9.97 (s, 2H), 8.63 (dd, *J* = 5.1, 1.0 Hz, 1H), 8.51 (dd, *J* = 8.4, 1.0 Hz, 1H), 7.82-7.76 (m, 4H), 7.75-7.69 (m, 2H), 7.63-7.57 (m, 4H), 7.33 (dd, *J* = 8.4, 0.7 Hz, 1H), 7.24 (dd, *J* = 7.8, 0.7 Hz, 1H) ppm; ^13^C NMR (125 MHz, CDCl_3_): δ_C_ 192.8, 158.2, 139.7, 139.5, 137.4, 135.5, 133.2, 132.3, 129.1, 128.6, 123.1, 116.5, 113.1, 110.2 ppm; ^11^B NMR (160 MHz, CDCl_3_): δ_B_ 11.8 ppm; HRMS-EI *m/z* [M]^+^ calcd for C_23_H_16_NO_3_B, 365.1223, found 365.1233.

**Di-(4-benzoylphenyl)borinic acid 8-hydroxyquinoline ester (3i)**

274 mg, 53%; yellow solid; mp 160-161 °C; ^1^H NMR (500 MHz, CDCl_3_) δ_H_ 8.65 (dd, *J* = 5.0, 1.0 Hz, 1H), 8.49 (dd, *J* = 8.4, 1.0 Hz, 1H), 7.83-7.76 (m, 4H), 7.76-7.68 (m, 6H), 7.61-7.52 (m, 6H), 7.44 (dd, *J* = 8.3, 7.1 Hz, 4H), 7.32 (d, *J* = 8.3 Hz, 1H), 7.25 (d, *J* = 7.7 Hz, 1H) ppm; ^13^C NMR (125 MHz, CDCl_3_): δ_C_ 197.1, 158.4, 139.5, 137.9, 137.5, 136.4, 133.2, 132.2, 131.7, 130.1, 129.4, 128.6, 128.2, 123.0, 112.9, 110.2 ppm; ^11^B NMR (160 MHz, CDCl_3_): δ_B_ 12.7 ppm; HRMS-EI *m/z* [M]^+^ calcd for C_35_H_24_NO_3_B, 517.1849, found 517.1842.

**Di-(4-cyanophenyl)borinic acid 8-hydroxyquinoline ester (3j)**

212 mg, 59%; mp 172-173 °C; ^1^H NMR (500 MHz, CDCl_3_) δ_H_ 8.57 (dd, *J* = 5.1, 1.0 Hz, 1H), 8.53 (dd, *J* = 8.3, 1.0 Hz, 1H), 7.77-7.68 (m, 2H), 7.57-7.52 (m, 4H), 7.52-7.47 (m, 4H), 7.35 (dd, *J* = 8.4, 0.6 Hz, 1H), 7.23 (dd, *J* = 7.8, 0.7 Hz, 1H) ppm; ^13^C NMR (126 MHz, CDCl_3_) δ_C_ 157.9, 139.9, 139.3, 137.4, 133.3, 132.3, 131.3, 128.6, 123.1, 119.4, 113.3, 110.1, 110.4 ppm; ^11^B NMR (160 MHz, CDCl_3_): δ_B_ 11.4 ppm; HRMS-EI *m/z* [M]^+^ calcd for C_23_H_14_N_3_OB, 359.1230, found 359.1223.

**Di-(3,4-methylenedioxyphenyl)borinic acid 8-hydroxyquinoline ester (3k)**

238 mg, 53%; yellow solid; mp 174-175 °C; ^1^H NMR (500 MHz, CDCl_3_) δ_H_ 8.51 (dd, *J* = 5.1, 1.0 Hz, 1H), 8.40 (dd, *J* = 8.4, 1.1 Hz, 1H), 7.66 (t, *J* = 8.0 Hz, 1H), 7.61 (dd, *J* = 8.3, 5.0 Hz, 1H), 7.25 (d, *J* = 8.3 Hz, 1H), 7.17 (d, *J* = 7.7 Hz, 1H), 6.90 (d, *J* = 8.4 Hz, 4H), 6.76 (d, *J* = 7.6 Hz, 2H), 5.87 (d, *J* = 1.8 Hz, 4H) ppm; ^13^C NMR (125 MHz, CDCl_3_): δ_C_ 158.5, 147.2, 146.7, 139.3, 138.8, 137.4, 133.0, 128.4, 125.3, 122.8, 112.3, 112.0, 109.8, 108.1, 100.2 ppm; ^11^B NMR (160 MHz, CDCl_3_): δ_B_ 12.1 ppm; HRMS-EI *m/z* [M]^+^ calcd for C_23_H_16_NO_5_B, 397.1122, found 397.1122.

**Di-(3,5-difluorophenyl)borinic acid 8-hydroxyquinoline ester (3l)**

267 mg, 70%; yellow solid; mp 191-192°C; ^1^H NMR (500 MHz, CDCl_3_) δ_H_ 8.55 (d, *J* = 5.1 Hz, 1H), 8.51 (d, *J* = 8.3 Hz, 1H), 7.78-7.65 (m, 2H), 7.34 (d, *J* = 8.3 Hz, 1H), 7.23 (d, *J* = 7.7 Hz, 1H), 6.95-6.80 (m, 4H), 6.71-6.60 (m, 2H) ppm; ^13^C NMR (125 MHz, CDCl_3_): δ_C_ 164.0 (d, *J* = 44.15 Hz), 162.0 (d, *J* = 11.44 Hz), 139.8, 139.4, 137.3, 133.2, 128.5, 123.0, 113.9, 113.8, 113.7 (d, *J* = 4.42 Hz), 113.2, 102.7, 102.5, 102.3 ppm; ^11^B NMR (160 MHz, CDCl_3_): δ_B_ 11.1 ppm; HRMS-EI *m/z* [M]^+^ calcd for C_21_H_12_F_4_NOB, 381.0948, found 381.0948.

**Di-(2-fluoro-5-methylphenyl)borinic acid 8-hydroxyquinoline ester (3m)**

299 mg, 80%); yellow solid; mp 179-180 °C; ^1^H NMR (500 MHz, CDCl_3_) δ_H_ 8.93 (dd, *J* = 5.2, 1.0 Hz, 1H), 8.38 (dd, *J* = 8.3, 1.0 Hz, 1H), 7.68-7.55 (m, 2H), 7.32 (dd, *J* = 6.8, 2.4 Hz, 2H), 7.24 (d, *J* = 8.3 Hz, 1H), 7.19 (d, *J* = 7.7 Hz, 1H), 7.02 (ddd, *J* = 7.9, 5.2, 2.4 Hz, 2H), 6.80 (dd, *J* = 9.8, 8.2 Hz, 2H), 2.27 (s, 6H) ppm; ^13^C NMR (125 MHz, CDCl_3_): δ_C_ 165.2, 163.3, 141.4 (t, *J* = 31.6 Hz), 138.9, 137.9, 135.3 (d, *J* = 39.75 Hz), 132.7, 132.4, 129.6 (d, 32.25 Hz), 128.6, 122.8, 114.5, 114.3, 112.6, 109.5, 20.8 ppm; ^11^B NMR (160 MHz, CDCl_3_): δ_B_ 11.5 ppm; HRMS-EI *m/z* [M]^+^ calcd for C_23_H_18_NOBF_2_, 373.1450, found 373.1444.

**Di-(6-methoxynaphthalen-2-yl)borinic acid 8-hydroxyquinoline ester (3n)**

399 mg, 85%; yellow solid; mp 249-250 °C; ^1^H NMR (500 MHz, CDCl_3_) δ_H_ 8.65 (dd, *J* = 5.1, 1.0 Hz, 1H), 8.42 (dd, *J* = 8.3, 1.0 Hz, 1H), 7.84 (d, *J* = 1.2 Hz, 2H), 7.74-7.61 (m, 6H), 7.58 (dd, *J* = 8.2, 1.4 Hz, 2H), 7.31-7.20 (m, 2H), 7.13-7.03 (m, 4H), 3.90 (s, 6H) ppm; ^13^C NMR (125 MHz, CDCl_3_): δ_C_ 153.6, 152.1, 134.2, 133.6, 132.5, 128.8, 127.8, 125.9, 125.6, 124.4, 123.7, 123.3, 120.7, 117.6, 112.8, 107.1, 104.7, 100.4, 50.1 ppm; ^11^B NMR (160 MHz, CDCl_3_): δ_B_ 12.6 ppm; HRMS-EI *m/z* [M]^+^ calcd for C_31_H_24_NO_3_B, 469.1849, found 469.1847.

**Di-(benzo[*b*]thiophen-2-yl)borinic acid 8-hydroxyquinoline ester** **(3o)**

320 mg, 76%; yellow solid; mp 204-206 °C (lit.^3^ 204-206 °C); ^1^H NMR (500 MHz, CDCl_3_) δ_H_ 8.70 (dd, *J* = 5.1, 1.0 Hz, 1H), 8.44 (dd, *J* = 8.3, 1.0 Hz, 1H), 7.81 (dd, *J* = 8.0, 1.1 Hz, 2H), 7.76-7.69 (m, 3H), 7.64 (dd, *J* = 8.3, 5.1 Hz, 1H), 7.52 (d, *J* = 0.8 Hz, 2H), 7.35-7.20 (m, 6H) ppm; ^13^C NMR (125 MHz, CDCl_3_): δ_C_ 158.0, 142.5, 141.4, 140.0, 139.6, 136.9, 133.1, 128.4, 127.2, 123.7, 123.5, 123.2, 123.0, 122.3, 113.2, 110.5; HRMS-EI *m/z* [M]^+^ calcd for C_25_H_16_NOS_2_B, 421.0766, found 421.0758.

**Di-(4-vinylphenyl)borinic acid 8-hydroxyquinoline ester (3p)**

238 mg, 66%; yellow solid; mp 197-198 °C; ^1^H NMR (500 MHz, CDCl_3_) δ_H_ 8.56 (dd, *J* = 5.0, 1.1 Hz, 1H), 8.40 (dd, *J* = 8.3, 1.0 Hz, 1H), 7.67 (t, *J* = 8.0 Hz, 1H), 7.61 (dd, *J* = 8.2, 5.0 Hz, 1H), 7.45-7.38 (m, 4H), 7.37-7.30 (m, 4H), 7.25 (d, *J* = 8.3 Hz, 1H), 7.19 (d, *J* = 7.7 Hz, 1H), 6.70 (dd, *J* = 17.6, 10.9 Hz, 2H), 5.71 (dd, *J* = 17.6, 1.2 Hz, 2H), 5.17 (dd, *J* = 10.9, 1.1 Hz, 2H) ppm; ^13^C NMR (125 MHz, CDCl_3_): δ_C_ 158.7, 139.2, 138.8, 137.6, 137.2, 136.3, 133.0, 132.2, 128.5, 125.5, 122.8, 112.9, 112.3, 109.8 ppm; ^11^B NMR (160 MHz, CDCl_3_): δ_B_ 12.8 ppm; HRMS-EI *m/z* [M]^+^ calcd for C_25_H_20_NOB, 361.1638, found 361.1642.

**Divinyl borinic acid 8-hydroxyquinoline ester** **(3q)**

55 mg, 26%; oil; ^1^H NMR (500 MHz, CDCl_3_) δ_H_ 8.42-8.31 (m, 2H), 7.69-7.54 (m, 2H), 7.20 (dd, *J* = 8.3, 0.7 Hz, 1H), 7.07 (dd, *J* = 7.7, 0.7 Hz, 1H), 6.31 (dd, *J* = 19.4, 13.3 Hz, 2H), 5.53 (dd, *J* = 13.3, 3.8 Hz, 2H), 5.35 (dd, *J* = 19.4, 3.9 Hz, 2H) ppm; ^13^C NMR (125 MHz, CDCl_3_): δ_C_ 153.5, 133.6, 133.2, 132.3, 127.6, 123.3, 117.3, 117.2, 106.7, 104.3 ppm; ^11^B NMR (160 MHz, CDCl_3_): δ_B_ 13.4 ppm; HRMS-EI *m/z* [M]^+^ calcd for C_13_H_12_NOB, 209.1012, found 209.1005.

**Di-(*E*-1-penten-1-yl)borinic acid 8-hydroxyquinoline ester (3r)**

117 mg, 40%; oil; ^1^H NMR (500 MHz, CDCl_3_) 8.35-8.31 (m, 2H), 7.61-7.56 (m, 2H), 7.16 (d, *J* = 7.7Hz, 1H), 7.03 (d, *J* = 7.7 Hz, 1H), 5.87-5.63 (m, 4H), 2.02 (qd, *J* = 7.0, 3.1 Hz, 4H), 1.37 (h, *J* = 7.4 Hz, 4H), 0.86 (t, *J* = 7.4 Hz, 6H) ppm; ^13^C NMR (125 MHz, CDCl_3_): δ_C_ 158.8, 138.7, 138.6, 137.9, 137.5, 132.6, 128.5, 122.4, 114.5, 109.3, 37.9, 22.4, 13.8 ppm; ^11^B NMR (160 MHz, CDCl_3_): δ_B_ 11.0 ppm; HRMS-EI *m/z* [M]^+^ calcd for C_19_H_24_NOB, 293.1951, found 293.1947.

**Di-(*E*-2-cyclohexylvinyl)borinic acid 8-hydroxyquinoline ester (3s)**

179 mg, 48%; oil; ^1^H NMR (500 MHz, CDCl_3_) δ_H_ 8.32 (dd, *J* = 8.3, 1.1 Hz, 1H), 8.29 (dd, *J* = 5.0, 1.0 Hz, 1H), 7.64-7.53 (m, 2H), 7.16 (d, *J* = 8.2 Hz, 1H), 7.03 (d, *J* = 7.7 Hz, 1H), 5.77-5.60 (m, 4H), 1.89 (dddt, *J* = 11.7, 8.9, 6.0, 3.3 Hz, 2H), 1.67 (tdd, *J* = 10.2, 8.7, 6.6, 3.2 Hz, 8H), 1.60 (dddt, *J* = 9.9, 5.2, 3.5, 1.6 Hz, 2H), 1.22 (qt, *J* = 12.5, 3.6 Hz, 4H), 1.11 (tt, *J* = 12.3, 3.2 Hz, 2H), 1.08-0.97 (m, 4H) ppm; ^13^C NMR (125 MHz, CDCl_3_): δ_C_ 158.8, 144.6, 138.6, 137.9, 137.5, 132.6, 128.4, 122.4, 111.4, 109.2, 42.9, 33.0, 26.4, 26.3 ppm; ^11^B NMR (160 MHz, CDCl_3_): δ_B_ 11.4 ppm; HRMS-EI *m/z* [M]^+^ calcd for C_25_H_32_NOB, 373.2577, found 373.2567.

**Di-(*E*-styryl)borinic acid 8-hydroxyquinoline ester (3t)**

285 mg, 79%; yellow solid; mp 144-146 °C; ^1^H NMR (500 MHz, CDCl_3_) δ_H_ 8.50 (dd, *J* = 5.0, 1.1 Hz, 1H), 8.41 (dd, *J* = 8.3, 1.1 Hz, 1H), 7.70-7.60 (m, 2H), 7.45-7.39 (m, 4H), 7.30-7.22 (m, 7H), 7.19-7.12 (m, 3H), 6.75 (d, *J* = 1.7 Hz, 4H) ppm; ^13^C NMR (125 MHz, CDCl_3_): δ_C_ 158.8, 139.3, 138.9, 138.5, 137.4, 132.8, 128.6, 128.3, 126.9, 126.3, 122.7, 112.1, 109.7 ppm; ^11^B NMR (160 MHz, CDCl_3_): δ_B_ 9.40 ppm; HRMS-EI *m/z* [M]^+^ calcd for C_25_H_20_NOB, 361.1638, found 361.1633.

**B. X-Ray Crystal Structures**

**B1. X-Ray crystal structure of 3f**

Crystals were grown by slow diffusion of hexanes into an acetone solution of **3f** and the X-ray crystal structure was deposited in the Cambridge Crystallographic Data Centre CCDC 1430431.


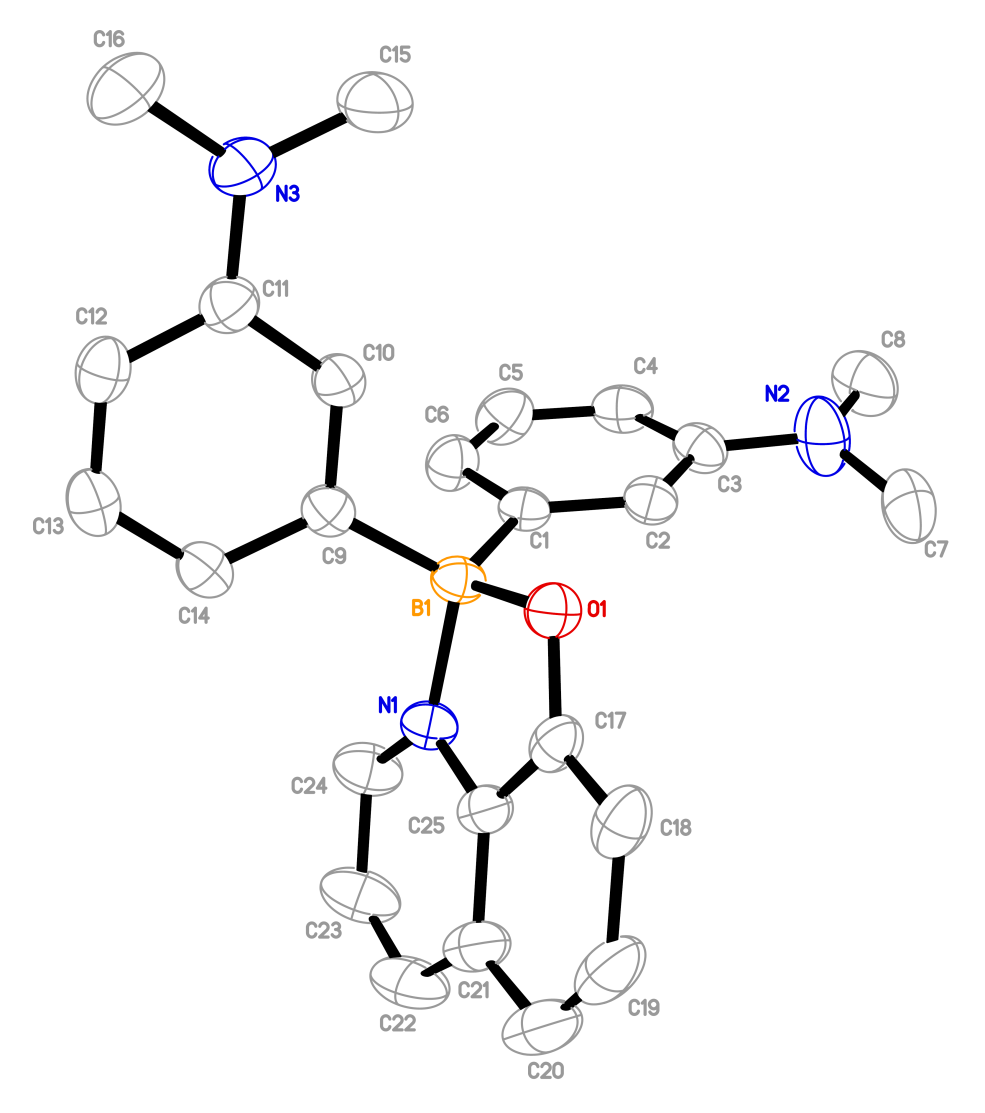


**B2. X-Ray crystal structure of 3h**

Crystals were grown by slow diffusion of hexanes into an acetone solution of **3h** and X-ray crystal structure was deposited in the Cambridge Crystallographic Data Centre CCDC 1472400.


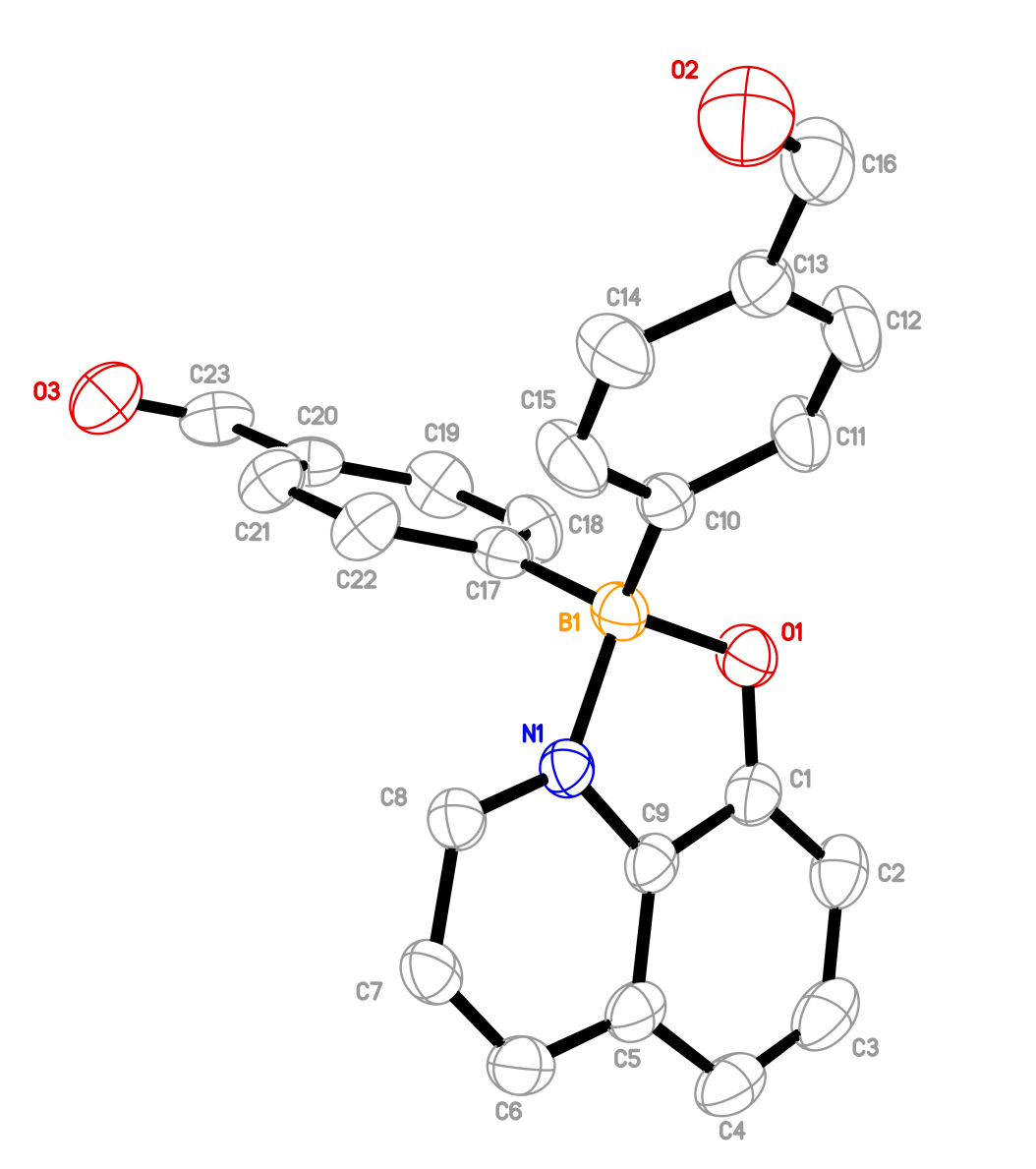


**B3. X-Ray crystal structure of 3o**

Crystals were grown by slow diffusion of hexanes into an acetone solution of **3o** and the X-ray crystal structure was deposited in the Cambridge Crystallographic Data Centre CCDC 1015712.

**
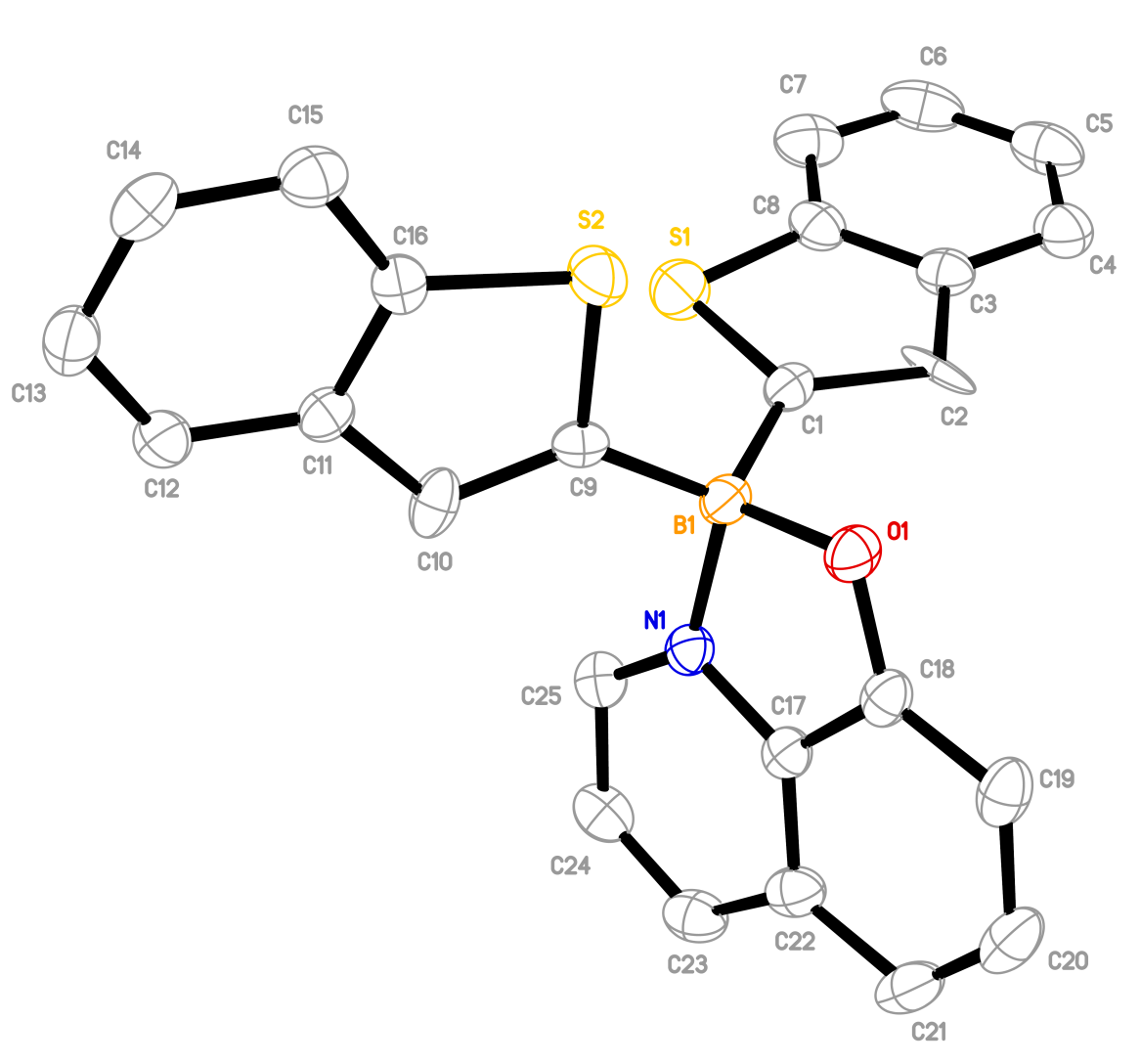
**

**C. Various *N,O*-type Organoborons**

**General Procedure for the preparation of 4a**

To a 100 mL round bottomed flask equipped with magnetic stirring bar and reflux condenser, were added sequentially 2-pyridineethanol (123.2 mg, 1.0 mmol), PhB(OH)_2_ (1.12 g, 9.2 mmol), K_3_PO_4_ (636.5 mg, 3.0 mmol) and 1,4-dioxane (50 mL). The mixture was refluxed for 20 h, and then the solvent was evaporated under reduced pressure. The resulting crude product was taken up in EtOAc (20 mL) and water (20 mL). The separated organic layer was successively washed with 10% aq. K_3_PO_4_ solution (3 x 10 mL) and brine (10 mL), dried over anhydrous Na_2_SO_4_, and evaporated to dryness under reduced pressure. The residue was purified by column chromatography on silica gel (EtOAc/hexanes = 1/9) to afford **4a** (172 mg, 60%) as a white solid.

**2-(2-((Diphenylboryl)oxy)ethyl)pyridine (4a)**

mp 163-164 °C (lit.^4^ 162-169 °C); ^1^H NMR (500 MHz, CDCl_3_) δ_H_ 8.22 (dd, *J* = 5.9, 1.7 Hz, 1H), 7.90 (td, *J* = 7.7, 1.6 Hz, 1H), 7.42 (d, *J* = 7.9 Hz, 1H), 7.31 (t, *J* = 6.8 Hz, 1H), 7.28-7.16 (m, 10H), 4.09 (t, *J* = 5.9 Hz, 2H), 3.19 (t, *J* = 5.9 Hz, 2H) ppm; ^13^C NMR (125 MHz, CDCl_3_): δ_C_ 157.90, 145.6, 139.8, 133.1, 127.2, 126.85, 126.2, 122.0, 57.8, 35.5 ppm; HRMS-EI *m/z* [M]^+^ calcd for C_19_H_18_NOB, 287.1481, found 287.1487.

**2-((4-Chlorophenyl)((diphenylboranyl)oxy)methyl)pyridine (4b)**

249 mg, 65%; white solid; mp 138-140 °C; ^1^H NMR (500 MHz, CDCl_3_) δ_H_ 8.46 (dt, *J* = 5.6, 1.2 Hz, 1H), 7.94 (td, *J* = 7.7, 1.4 Hz, 1H), 7.55-7.50 (m, 5H), 7.44-7.37 (m, 4H), 7.29 (dddd, *J* = 12.7, 6.0, 3.3, 1.6 Hz, 5H), 7.25-7.21 (m, 2H), 6.11 (s, 1H) ppm; ^13^C NMR (125 MHz, CDCl_3_): δ_C_ 161.0, 140.9 (d, *J* = 19.50 Hz), 139.6, 134.4, 133.4, 132.1, 129.0 (d, *J* = 23.30 Hz), 127.4 (d, *J* = 39.0), 127.0, 126.3, 124.2, 121.20, 80.2 ppm; ^11^B NMR (160 MHz, CDCl_3_): δ_B_ 11.1 ppm; HRMS-EI *m/z* [M]^+^ calcd for C_24_H_19_ClNOB, 383.1248, found 383.1241.

**2-(2-((Diphenylboryl)oxy)propan-2-yl)pyridine (4c)**

151 mg, 50%; white solid; mp 212-213 °C; ^1^H NMR (500 MHz, CDCl_3_) δ_H_ 8.39 (dt, *J* = 5.7, 1.2 Hz, 1H), 8.04 (td, *J* = 7.7, 1.4 Hz, 1H), 7.53-7.48 (m, 2H), 7.42-7.34 (m, 4H), 7.26-7.21 (m, 4H), 7.21-7.16 (m, 2H), 1.64 (s, 6H) ppm; ^13^C NMR (125 MHz, CDCl_3_): δ_C_ 165.8, 141.2, 140.8, 135.7, 132.5, 128.0, 127.2, 126.2, 123.9, 119.9, 80.6, 30.2 ppm; ^11^B NMR (160 MHz, CDCl_3_): δ_B_ 10.6 ppm; HRMS (EI) *m/z* [M]^+^ calcd for C_20_H_20_NOB, 301.1638, found 301.1641.

**2-((Diphenylboryl)oxy)-N,N-dimethylaniline** **(4d)**

160 mg, 53%; white solid; mp 164-165 °C; ^1^H NMR (500 MHz, CDCl_3_) δ_H_ 7.78-7.69 (m, 4H), 7.36-7.27 (m, 5H), 7.27-7.22 (m, 2H), 7.21 (dd, *J* = 8.1, 1.2 Hz, 1H), 7.11 (dd, *J* = 7.9, 1.4 Hz, 1H), 6.86 (td, *J* = 7.7, 1.2 Hz, 1H), 2.74 (s, 6H) ppm; ^13^C NMR (125 MHz, CDCl_3_): δ_C_ 157.5, 138.8, 133.0, 132.9, 130.1, 127.4, 127.34, 126.9, 118.4, 117.4, 114.5, 50.4 ppm; ^11^B NMR (160 MHz, CDCl_3_): δ_B_ 9.1 ppm; HRMS-EI *m/z* [M]^+^ calcd for C_20_H_20_NOB, 301.1638, found 301.1620.

**((Diphenylboryl)oxy)(pyridin-2-yl)methanone** **(4e)**

98 mg, 34%; white solid; mp 158-159 °C; ^1^H NMR (500 MHz, CDCl_3_) δ_H_ 8.71 (dt, *J* = 5.6, 1.2 Hz, 1H), 8.42-8.32 (m, 2H), 7.91 (dq, *J* = 5.5, 4.2 Hz, 1H), 7.38 (dq, *J* = 7.3, 2.4 Hz, 4H), 7.32-7.26 (m, 6H) ppm; ^13^C NMR (125 MHz, CDCl_3_): δ_C_ 163.3, 143.4, 143.2, 141.8, 132.18, 128.9, 127.8, 127.6, 123.85 ppm; ^11^B NMR (160 MHz, CDCl_3_): δ_B_ 10.2 ppm; HRMS-EI *m/z* [M]^+^ calcd for C_18_H_14_NO_2_B, 287.1118, found 287.1109.

**Diphenyl borinic acid (L)-proline ester** **(4f)**

148 mg, 53%; white solid; mp 272-273 °C; ^1^H NMR (500 MHz, *d*_6_-DMSO): δ_H_ 7.91 (d, *J* = 6.3 Hz, 1H), 7.48-7,41 (m, 4H), 7.26-7.12 (m, 6H), 4.26-4.19 (m, 1H), 3.04-2.94 (m, 1H), 2.49-2.35 (m, 1H), 2.04-1.96 (m, 2H), 1.78-1.77 (m, 2H) ppm; ^13^C NMR (125 MHz, DMSO-*d*_6_): δ_C_ 175.1, 131.7, 131.4, 127.7, 127.6, 62.7, 50.6, 27.8, 25.3 ppm; ^11^B NMR (160 MHz, CDCl_3_): δ_B_ 7.6 ppm; HRMS-EI *m/z* [M]^+^ calcd for C_17_H_18_NO_2_B, 279.1431, found 279.1440.

**2-(2-((Diphenylboryl)oxy)phenyl)pyridine (4g)**

292 mg, 87%; white solid; mp 203-204 °C (lit.^5^ 200 °C); ^1^H NMR (500 MHz, CDCl_3_) δ_H_ 8.14 (ddd, *J* = 6.0, 1.7, 0.8 Hz, 1H), 8.05-7.94 (m, 2H), 7.62 (dd, *J* = 8.0, 1.6 Hz, 1H), 7.41-7.31 (m, 2H), 7.28-7.24 (m, 6H), 7.24-7.15 (m, 7H), 6.84 (ddd, *J* = 8.3, 7.2, 1.2 Hz, 1H) ppm; ^13^C NMR (125 MHz, CDCl_3_): δ_C_ 160.1, 150.9, 144.1, 134.3, 133.3, 127.3, 126.5, 125.4, 121.7, 121.2, 120.7, 119.3, 118.4 ppm; HRMS-EI *m/z* [M]^+^ calcd for C_23_H_18_NOB, 335.1481, found 335.1471.

**2-(5-((Diphenylboryl)oxy)-3-phenyl-1H-pyrazol-1-yl)pyridine (4h)**

337 mg, 84%; white solid; mp 246-247 °C; ^1^H NMR (500 MHz, CDCl_3_) δ_H_ 8.18-8.04 (m, 2H), 7.93 (dd, *J* = 6.2, 1.6 Hz, 1H), 7.87-7.78 (m, 2H), 7.44-7.34 (m, 3H), 7.32-7.21 (m, 11H), 5.98 (s, 1H) ppm; ^13^C NMR (125 MHz, CDCl_3_): δ_C_ 157.06, 156.6, 147.0, 143.0, 142.1, 133.0, 132.5, 129.1, 128.6, 127.6, 127.2, 126.0, 119.3, 113.4, 86.6 ppm; ^11^B NMR (160 MHz, CDCl_3_): δ_B_ 7.8 ppm; HRMS-EI *m/z* [M]^+^ calcd for C_26_H_20_N_3_OB, 401.1699, found 401.1692.

**2-(2-((Diphenylboryl)oxy)phenyl)imidazo[1,2-a]pyridine (4i)**

333 mg, 89%; white solid; mp 283-285 °C; ^1^H NMR (500 MHz, CDCl_3_) δ_H_ 7.98 (dt, *J* = 6.8, 1.2 Hz, 1H), 7.44 (s, 1H), 7.40-7.31 (m, 4H), 7.26-7.13 (m, 10H), 6.94 (td, *J* = 6.9, 1.2 Hz, 1H), 6.88 (dd, *J* = 9.2, 1.0 Hz, 1H), 6.66 (td, *J* = 7.4, 1.3 Hz, 1H) ppm; ^13^C NMR (125 MHz, CDCl_3_): δ_C_ 158.2, 140.6, 137.8, 133.2, 131.8, 128.4, 127.3, 126.4, 126.1, 124.6, 120.6, 118.4, 115.1, 114.7, 114.2, 105.6 ppm; ^11^B NMR (160 MHz, CDCl_3_): δ_B_ 4.6 ppm; HRMS-EI *m/z* [M]^+^ calcd for C_25_H_19_N_2_OB, 374.1590, found 374.1587.

**5,7-Dichloro-8-((diphenylboranyl)oxy)quinoline (4j)**

329 mg, 87%; yellow solid; mp 209-210 °C; ^1^H NMR (500 MHz, CDCl_3_) δ_H_ 8.67 (dd, *J* = 5.1, 1.0 Hz, 1H), 8.64 (dd, *J* = 8.5, 1.0 Hz, 1H), 7.78-7.70 (m, 2H), 7.48-7.40 (m, 4H), 7.31-7.24 (m, 6H) ppm; ^13^C NMR (126 MHz, CDCl_3_) δ_C_ 153.8, 141.1, 137.8, 136.9, 132.6, 132.0, 127.7, 127.3, 125.21, 123.4, 116.0, 114.5 ppm; ^11^B NMR (160 MHz, CDCl_3_): δ_B_ 13.8 ppm; HRMS-EI *m/z* [M]^+^ calcd for C_21_H_14_NCl_2_OB, 377.0545, found 377.0537.

**2-(2-((Diphenylboryl)oxy)phenyl)benzo[d]oxazole (4k)**

308 mg, 82%; white solid; mp 193-194 °C; ^1^H NMR (500 MHz, CDCl_3_) δ_H_ 7.80 (dd, *J* = 7.9, 1.7 Hz, 1H), 7.66 (d, *J* = 8.3 Hz, 1H), 7.53 (ddd, *J* = 8.8, 7.2, 1.8 Hz, 1H), 7.47-7.40 (m, 5H), 7.30-7.20 (m, 8H), 7.00 (dd, *J* = 8.4, 1.1 Hz, 1H), 6.94-6.87 (m, 1H) ppm; ^13^C NMR (125 MHz, CDCl_3_): δ_C_ 163.2, 161.5, 149.2, 137.6, 133.3, 133.1, 127.4, 126.8, 126.7, 126.4, 126.0, 120.8, 119.0, 117.7, 111.4, 108.2 ppm; HRMS-EI *m/z* [M]^+^ calcd for C_25_H_18_NO_2_B, 375. 1431, found 375.1429.

**2-(2-((Diphenylboryl)oxy)phenyl)benzo[d]thiazole (4l)**

344 mg, 88%; white solid; mp 215-216 °C; ^1^H NMR (500 MHz, CDCl_3_) δ_H_ 7.86 (dd, *J* = 8.2, 1.0 Hz, 1H), 7.52 (dd, *J* = 7.9, 1.6 Hz, 1H), 7.49-7.34 (m, 6H), 7.31-7.15 (m, 9H), 6.88-6.80 (m, 1H); ^13^C NMR (125 MHz, CDCl_3_): δ_C_ 168.6, 159.6, 146.2, 136.8, 133.3, 133.2, 130.1, 127.7, 127.4, 126.7, 126.2, 121.9, 121.7, 120.9, 119.3, 115.6 ppm; HRMS-EI *m/z* [M]^+^ calcd for C_25_H_18_NOBS, 391. 1202, found 391.1210.

**8-((Bis(4-fluorophenyl)boranyl)oxy)-5,7-dichloroquinoline (4m)**

339 mg, 82%; yellow solid; mp 189-190 °C; ^1^H NMR (500 MHz, CDCl_3_) δ_H_ 8.67 (dq, *J* = 8.4, 1.6 Hz, 1H), 8.60 (dq, *J* = 4.6, 1.6 Hz, 1H), 7.82-7.70 (m, 2H), 7.40-7.31 (m, 4H), 6.97 (tt, *J* = 8.5, 1.8 Hz, 4H) ppm; ^13^C NMR (125 MHz, CDCl_3_) δ_C_ 163.7, 161.7, 153.5, 141.0, 137.6, 137.3, 133.7, 133.6, 132.7, 125.2, 123.5, 116.4, 114.7, 114.7, 114.5 ppm; HRMS-EI *m/z* [M]^+^ calcd for C_21_H_12_NCl_2_F_2_OB, 413. 0357, found 413.0359.

**2-(2-((Bis(4-fluorophenyl)boryl)oxy)phenyl)benzo[*d*]oxazole (4n)**

350 mg, 85%; white solid; mp 212-213 °C; ^1^H NMR (500 MHz, CDCl_3_) δ_H_ 7.82 (dd, *J* = 7.9, 1.7 Hz, 1H), 7.69 (dt, *J* = 8.4, 0.8 Hz, 1H), 7.56 (ddd, *J* = 8.7, 7.2, 1.8 Hz, 1H), 7.46 (ddd, *J* = 8.4, 7.4, 1.2 Hz, 1H), 7.41-7.34 (m, 4H), 7.31 (td, *J* = 7.8, 1.0 Hz, 1H), 7.20 (dd, *J* = 8.5, 1.0 Hz, 1H), 6.97-6.91 (m, 6H) ppm; ^13^C NMR (125 MHz, CDCl_3_): δ_C_ 163.5, 162.9, 161.5, 149.2, 137.8, 134.6, 134.51, 133.0, 126.8, 126.6, 126.0, 120.7, 119.4, 117.3, 114.3, 114.2, 116.7, 108.1 ppm; HRMS-EI *m/z* [M]^+^ calcd for C_25_H_16_NO_2_BF_2_, 411. 1242, found 411.1235.

**2-(2-((Bis(4-fluorophenyl)boranyl)oxy)phenyl)benzo[*d*]thiazole (4o)**

355 mg, 83%; yellow solid; mp 198-199 °C; ^1^H NMR (500 MHz, CDCl_3_) δ_H_ 7.89 (dd, *J* = 8.2, 1.2 Hz, 1H), 7.54 (dd, *J* = 7.8, 1.6 Hz, 1H), 7.51-7.42 (m, 2H), 7.35-7.28 (m, 5H), 7.22 (d, *J* = 8.6 Hz, 1H), 7.16 (dd, *J* = 8.5, 1.0 Hz, 1H), 6.95-6.84 (m, 5H) ppm; ^13^C NMR (125 MHz, CDCl_3_): δ_C_168.7, 163.4, 161.5, 159.3, 145.9, 136.9, 134.7, 134.6, 130.2, 127.9, 127.4, 126.5, 122.1, 121.3, 120.8, 119.6, 115.5, 114.3, 114.2 ppm; HRMS-EI *m/z* [M]^+^ calcd for C_25_H_16_NOF_2_SB, 427. 1014, found 427.1022.

**(*E*)-2,2'-(1,1,6,6-Tetraphenyl-2,5-dioxa-1,6-diborahex-3-ene-3,4-diyl)dipyridine** **(4p)**

Compound **4p** was prepared according to the general procedure in which 6 equiv of K_3_PO_4_ and 18 equiv of phenyboronic acid were used in 1,4-dioxane (100 mL).

239 mg, 44%; yellow solid; mp 197-198 °C; ^1^H NMR (500 MHz, CDCl_3_) δ_H_ 8.81 (d, *J* = 8.2 Hz, 1H), 8.41 (s, 1H), 8.03 (d, *J* = 8.6 Hz, 2H), 7.56-7.44 (m, 4H), 7.33-7.19 (m, 20H) ppm; ^13^C NMR (126 MHz, CDCl_3_): δ_C_ 140.78, 132.90, 132.33, 127.46, 127.17, 126.79, 126.54 ppm; HRMS-EI *m/z* [M]^+^ calcd for C_36_H_28_N_2_O_2_B_2_, 542.2337, found 542.2329.

**2,5-Bis(2-((diphenylboryl)oxy)phenyl)thiazolo[5,4-*d*]thiazole (4q)**

Compound **4q** was prepared according to the general procedure in which 6 equiv of K_3_PO_4_ and 18 equiv of phenyboronic acid were used in 1,4-dioxane (50 mL).

406 mg, 62%; yellow solid; mp 233-236 °C; ^1^H NMR (500 MHz, CDCl_3_) δ_H_ 7.77 (ddd, *J* = 8.9, 7.4, 1.7 Hz, 2H), 7.46 (ddd, *J* = 8.6, 7.2, 1.6 Hz, 1H), 7.44-7.28 (m, 24H), 7.20 (d, *J* = 8.3 Hz, 1H), 7.16 (dd, *J* = 7.9, 1.6 Hz, 1H) ppm; ^13^C NMR (125 MHz, CDCl_3_): δ_C_175.77, 154.31, 136.90, 133.43, 130.57, 127.79, 126.88, 125.72, 120.67, 119.91, 116.64 ppm; HRMS-EI *m/z* [M]^+^ calcd for C_40_H_28_N_2_O_2_B_2_S_2_, 654.1778, found 654.1769.

**IV. Mechanistic Considerations**

**A. Control Experiments**

**A1. Reaction of phenylboronic acid with 8-hydroxyquinoline**

To a 100 mL round bottomed flask equipped with magnetic stirring bar and reflux condenser, were added sequentially 8-hydroxyquinoline (145.2 mg, 1.0 mmol), PhB(OH)_2_ (1.10 g, 9.0 mmol), K_3_PO_4_ (636.8 mg, 3.0 mmol) and 1,4-dioxane (50 mL). The mixture was refluxed for 20 h, and then the solvent was evaporated under reduced pressure. The resulting crude product was taken up in EtOAc (20 mL) and water (20 mL). The separated water layer was acidified to pH 6 with 1*N* HCl, and then extracted with EtOAc (3 x 10 mL). The combined organic layers were washed with brine, dried over anhydrous Na_2_SO_4_, and evaporated to dryness under reduced pressure. The residue was purified by column chromatography on silica gel using 10-30% EtOAc/hexanes gradient to afford **3a** (251 mg, 0.81 mmol) and **2b** (540 mg, 1.73 mmol).

Triphenylboroxine **(2b)**

^1^H NMR (500 MHz, CDCl_3_): δ_H_ δ 8.33-8.26 (m, 2H), 7.68-7.60 (m, 1H), 7.60-7.51 (m, 2H) ppm; ^13^C NMR (125 MHz, CDCl_3_): δ_C_ 135.6, 132.7, 128.0 ppm; ^11^B NMR (160 MHz, CDCl_3_): δ_B_ 30.6 ppm; FT-IR (neat, ν_max_): 1603, 1442, 1367, 1450, 1311 cm^-1^. HRMS-EI *m/z* [M+H]^+^ calcd for C_18_H_15_B_3_O_3_, 312.1300, found 312.1294.

**^11^B NMR and FT-IR of 2b**

**^11^B NMR and FT-IR of PhB(OH)_2_ for comparison with 2b**

^11^B NMR (CDCl_3_, 160 MHz): δ 30.4 ppm.

**A2. Reaction of phenylboronic acid without 8-hydroxyquinoline**

To a 100 mL round bottomed flask equipped with magnetic stirring bar and reflux condenser, were added PhB(OH)_2_ (1.10 g, 9.0 mmol), K_3_PO_4_ (636.8 mg, 3.0 mmol) and 1,4-dioxane (50 mL). The mixture was refluxed for 20 h, and then the solvent was evaporated under reduced pressure. The resulting crude product was taken up in EtOAc (20 mL) and water (20 mL). The separated water layer was acidified to pH 6 with 1*N* HCl, and then extracted with EtOAc (3 x 10 mL). The combined organic layers were washed with brine, dried over anhydrous Na_2_SO_4_, and evaporated to dryness under reduced pressure. The residue was purified by column chromatography on silica gel using 10-30% EtOAc/hexanes gradient to afford **5** (67 mg, 0.34 mmol) and **2b** (743 mg, 2.38 mmol).

**Diphenyl borinic acid (5)**

^1^H NMR (500 MHz, CDCl_3_): δ_H_ 7.96 (d, *J* = 7.05 Hz, 2H), 7.87 (d, *J* = 7.05 Hz, 1H), 7.59-7.54 (m, 2H), 7.53-7.51 (m, 5H) 5.95 (br s, 1H) ppm; ^13^C NMR (125 MHz, CDCl_3_): δ_C_ 135.9, 134.7, 127.9 ppm; ^11^B NMR (160 MHz, CDCl_3_): δ_B_ 46.8 ppm; HRMS-EI *m/z* [M]^+^ calcd for C_12_H_11_OB, 182.0903, found 182.0963.

**^11^B NMR of 5**

**A3. Reaction in the presence of 1-naphthol instead of 8-hydroxyquinoline**

To a 100 mL round bottomed flask equipped with magnetic stirring bar and reflux condenser, were added sequentially 1-naphthol (144.2 mg, 1.0 mmol), PhB(OH)_2_ (1.10 g, 9.0 mmol), K_3_PO_4_ (636.8 mg, 3.0 mmol) and 1,4-dioxane (50 mL). The reaction mixture was refluxed for 20 h, and then the solvent was evaporated under reduced pressure. The crude product was diluted with EtOAc (20 mL) and water (20 mL). The separated water layer was acidified to pH 6 with 1*N* HCl, and then extracted with EtOAc (3 x 10 mL). The combined organic layers were washed with brine, dried over anhydrous Na_2_SO_4_, and evaporated to dryness under reduced pressure. The residue was purified by column chromatography on silica gel using 10-30% EtOAc/hexanes gradient to afford **5** (51 mg, 0.28 mmol) and **2b** (710 mg, 2.27 mmol).

**A4. Reaction of phenylboronic acid with 8-hydroxyquinoline in the presence of TEMPO**

To a 100 mL round bottomed flask equipped with magnetic stirring bar and reflux condenser, were added sequentially 8**-**hydroxyquinoline (145.2 mg, 1.0 mmol), PhB(OH)_2_ (1.10 g, 9.0 mmol), TEMPO (8.0 mg, 0.05 mmol), K_3_PO_4_ (636.8 mg, 3.0 mmol) and 1,4-dioxane (50 mL) under argon atmosphere. The reaction mixture was refluxed for 20 h, and then the solvent was evaporated under reduced pressure. The crude product was diluted with EtOAc (20 mL) and water (20 mL). The separated water layer was acidified to pH 6 with 1*N* HCl, and then extracted with EtOAc (3 x 10 mL). The combined organic layers were washed with brine, dried over anhydrous Na_2_SO_4_, and evaporated to dryness under reduced pressure. The residue was purified by column chromatography on silica gel using 10-30% EtOAc/hexanes gradient to afford **3a** (241 mg, 0.78 mmol) and **2b** (509 mg, 1.64 mmol).

**B. MALDI-TOF and ^11^B NMR Studies**

**B1. Observation of di(quinolin-8-yl) boronate I**

A stirred solution of 8-hydroxyquinoline (36.3 mg, 0.25 mmol) and PhB(OH)_2_ (274.3 mg, 2.25 mmol) in toluene (12.5 mL) was refluxed for 16 h. An aliquot from the reaction mixture was withdrawn and THF was added to make the sample homogeneous. MALDI-TOF showed the presence of di(quinolin-8-yl) boronate **I** at *m/z* = 377 [M+H]^+^ and a borenium cation at *m/z* = 232. While analyzing ^11^B NMR, an aliquot was withdrawn from the mixture and the solvent was evaporated in vacuo. The resulting solid was dissolved in benzene-*d_6_* (0.7 mL) and analyzed by ^11^B NMR. The NMR showed two peaks at 9.5 and 31.3 ppm, which correspond to the sp^3^-hybridized boron of **I** and sp^2^-hybridized boroxine **2b**, respectively. However, **I** could not be isolable.

**MALDI-TOF spectrum (without base)**


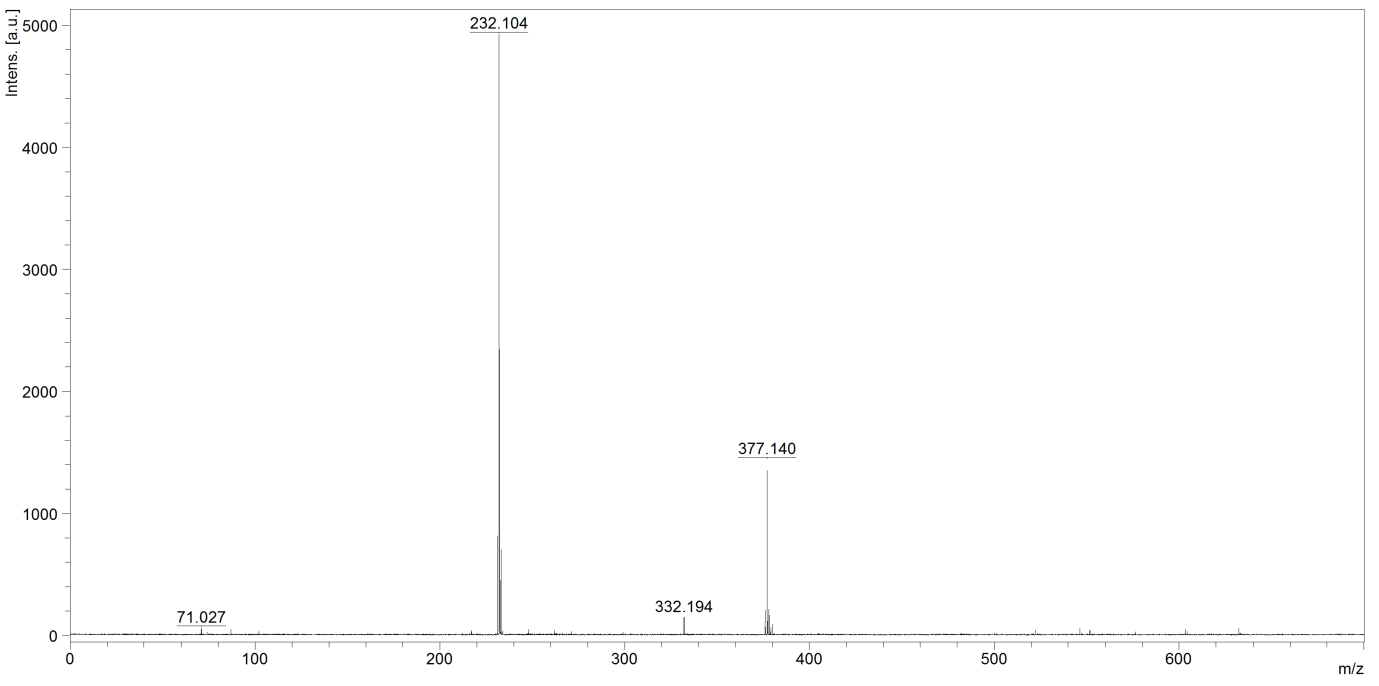


MALDI-TOF *m/z*: 232, 377 ([M+H]^+^).

**^11^B NMR (without base)**


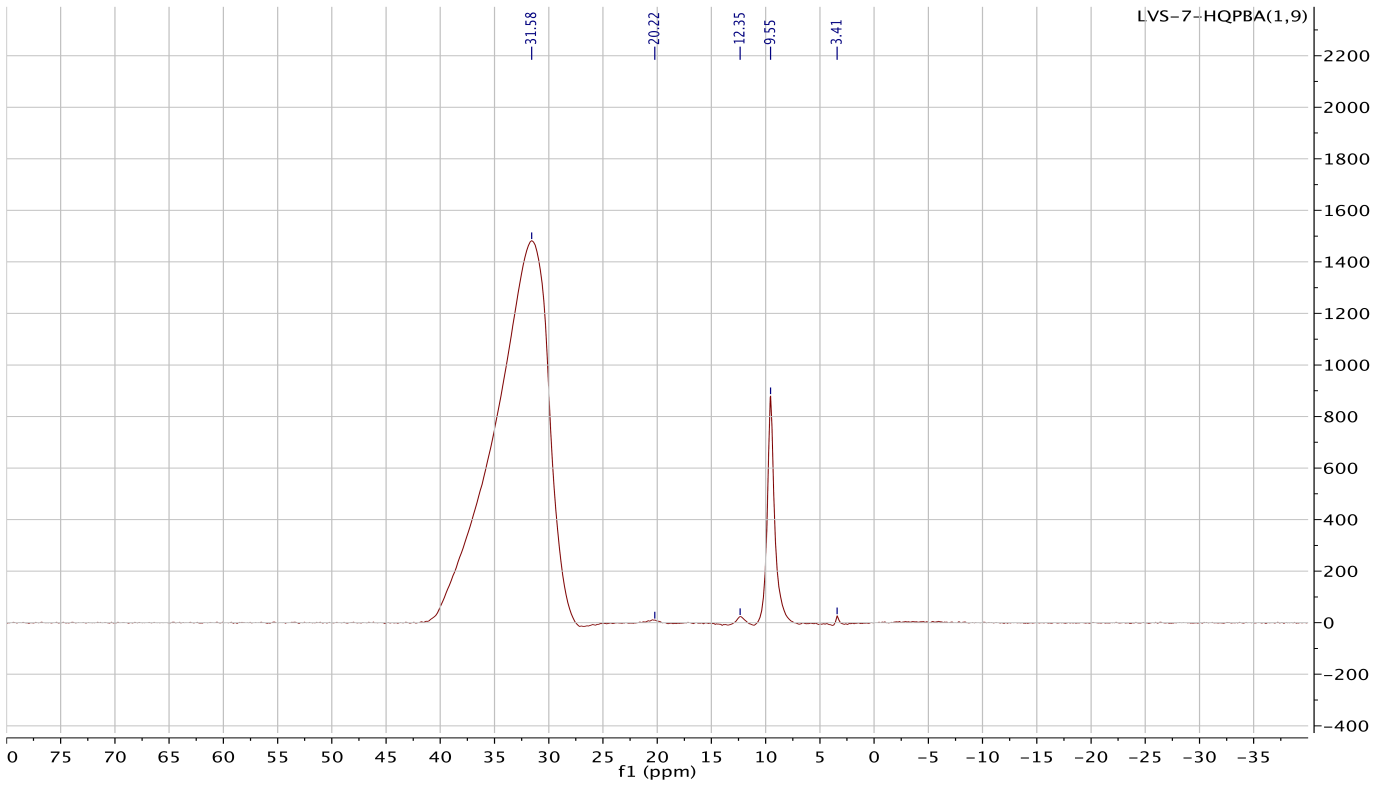


^11^B NMR (C_6_D_6_, 160 MHz): δ 31.5, 9.5 ppm.

**B2. Base-promoted reaction monitored by MALDI-TOF and ^11^B NMR**

**MALDI-TOF Analysis**

A stirred solution of 8-hydroxyquinoline (36.3 mg, 0.25 mmol) and PhB(OH)_2_ (274.3 mg, 2.25 mmol) in toluene (12.5 mL) was refluxed for 16 h. After that K_3_PO_4_ (159.2mg, 0.75 mmol) was added and the mixture was heated to reflux for 6 h. An aliquot was drawn from the reaction mixture at that time and THF was added to make the sample homogeneous, and analyzed by MALDI-TOF.

**MALDI-TOF spectrum obtained from the reaction mixture after 6 h (with base)**


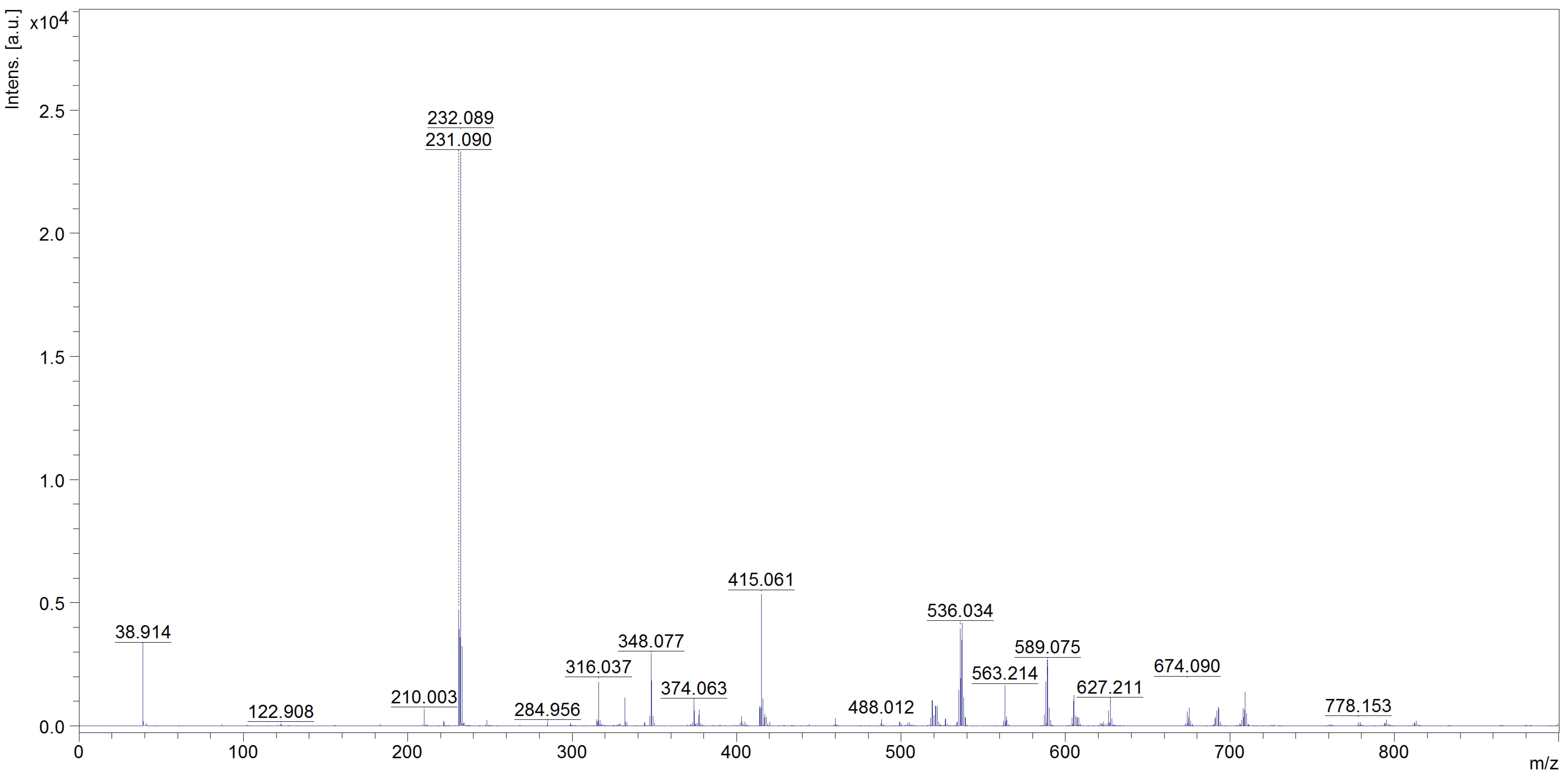


**Possible boron intermediates identified by MALDI-TOF**

**^11^B NMR Analysis**

To a stirred solution of 8-hydroxyquinoline (36.3 mg, 0.25 mmol) and PhB(OH)_2_ (274.3 mg, 2.75 mmol) in benzene-*d_6_* (12.5 mL), K_3_PO_4_ (159.2 mg, 0.75 mmol) was added, and then the mixtures were heated to reflux. The reaction was stopped and allowed the insoluble materials to settle by gravity while sampling. Aliquots were carefully withdrawn from the solution phase at different intervals of time using a syringe, and were analyzed by ^11^B NMR

**Analysis of the reaction mixture by ^11^B NMR at a given time (with base)**

After 5 minutes


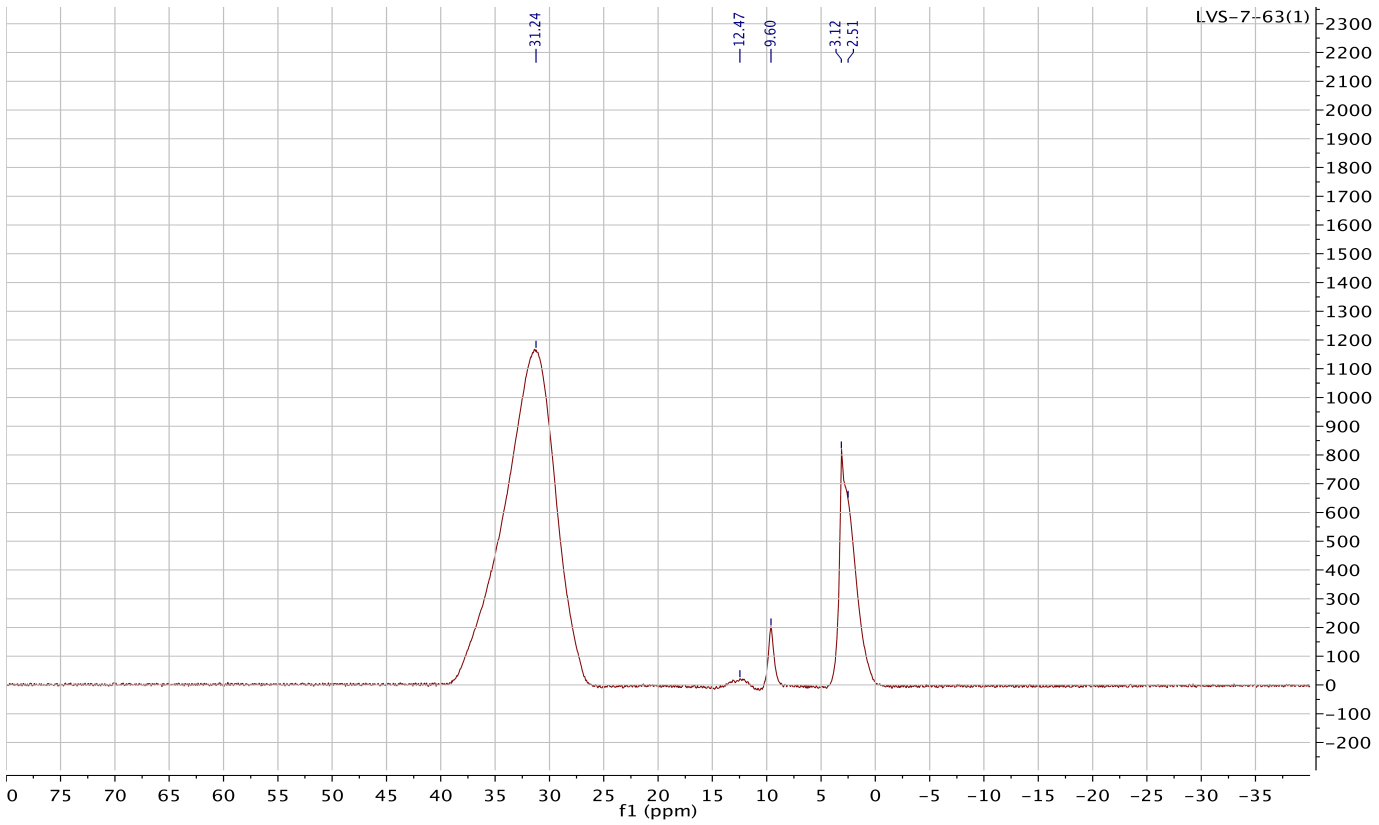


After 3 hours


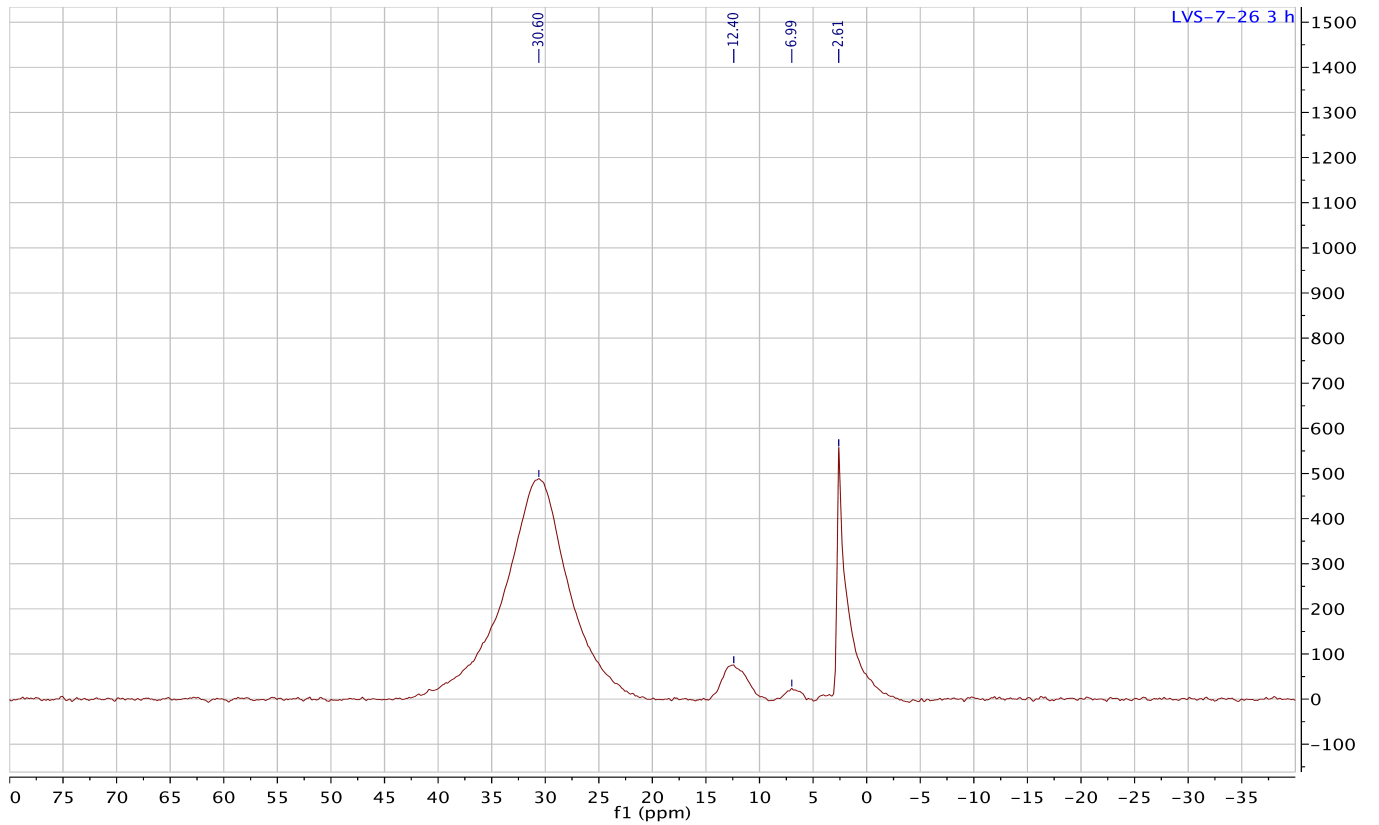


After 6 hours


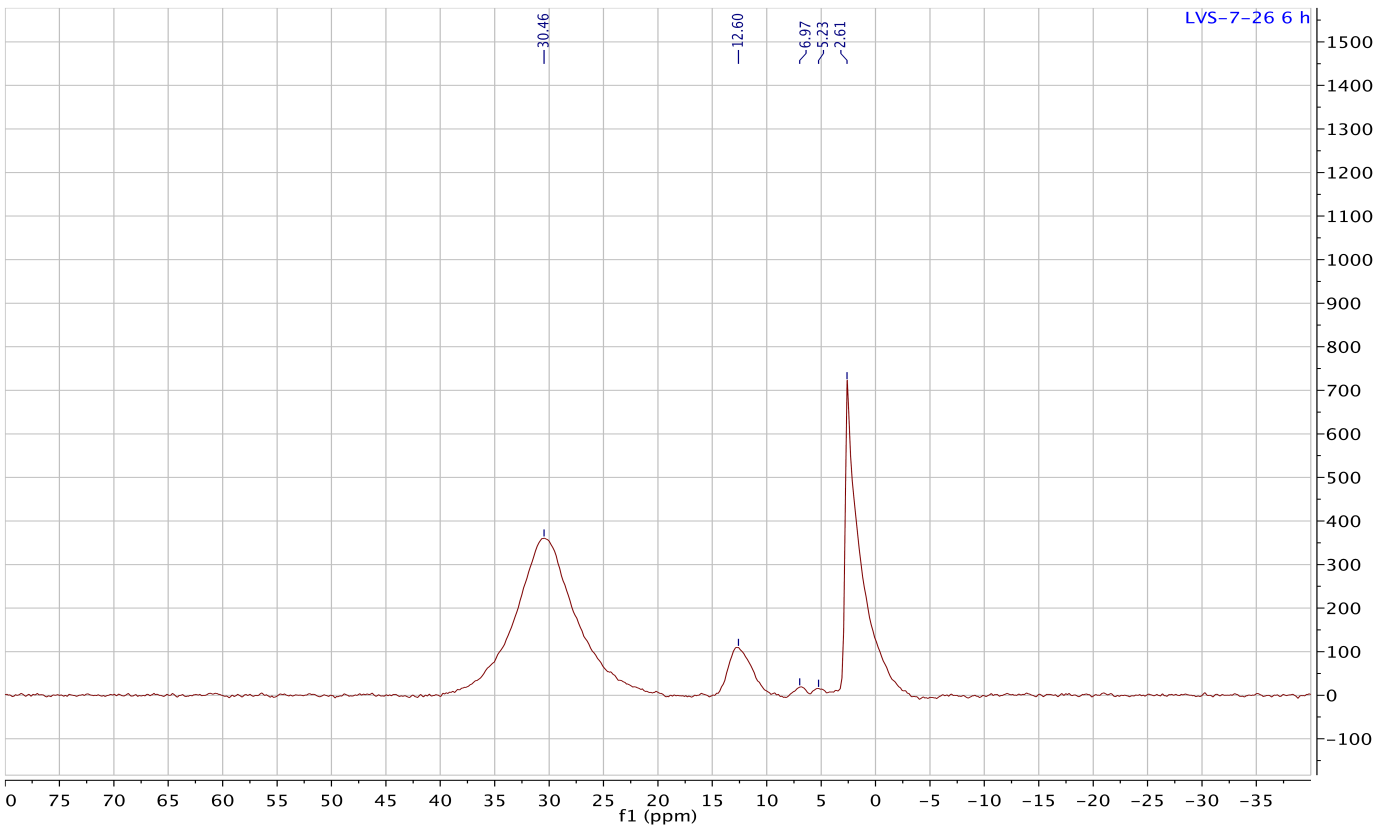


After 9 hours


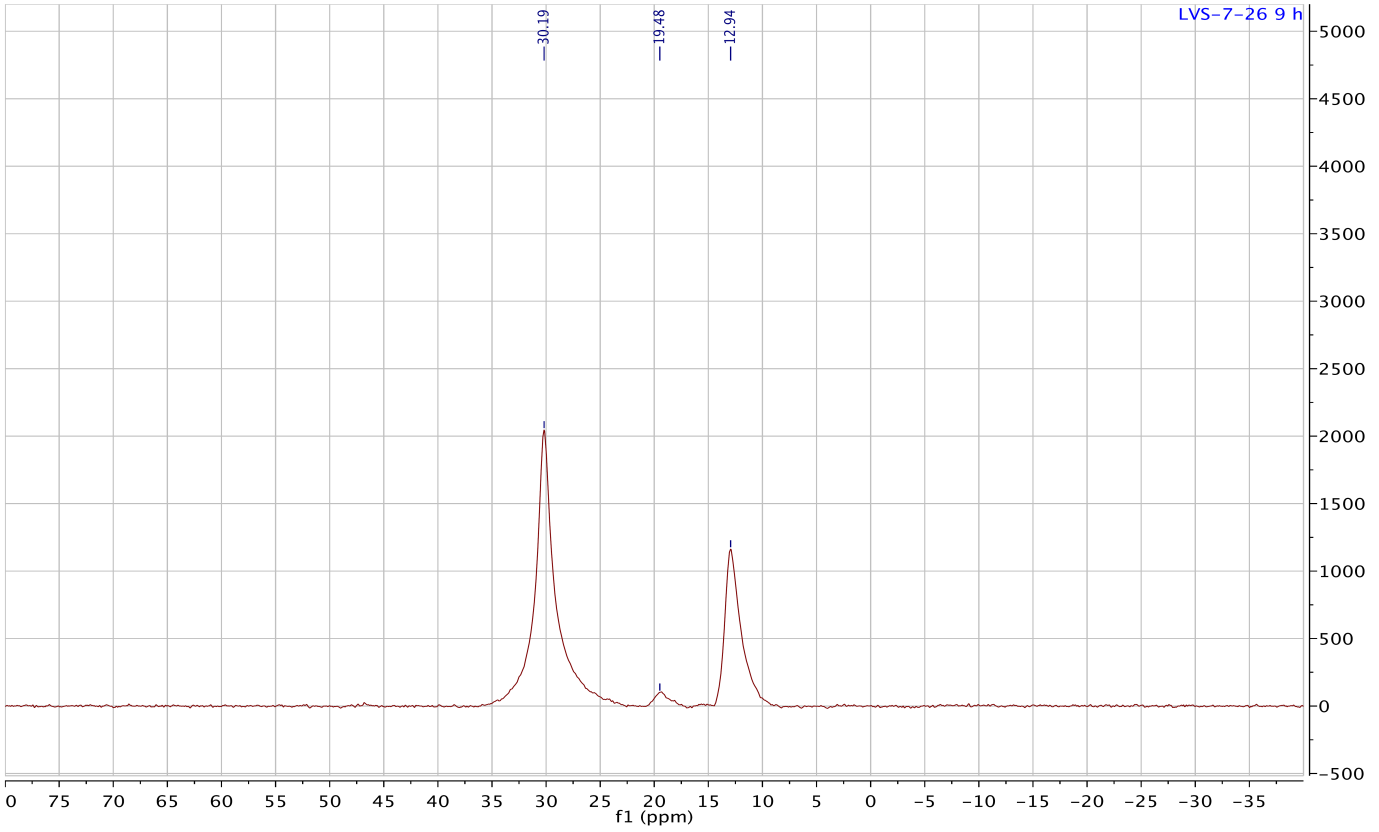


**V. Preparation of Four-Coordinate Organoborons from *O,O*-Ligands**

**General Procedure for the preparation of 6a**

To a 100 mL round bottomed flask equipped with magnetic stirring bar and reflux condenser, were added sequentially 1,3-diphenyl-1,3-propanedione (224.3 mg, 1.0 mmol), 4-iodophenylboronic acid (2.23 g, 9.0 mmol), K_3_PO_4_ ( 636.8 mg, 3.0 mmol) and 1,4-dioxane (50 mL). The mixture was refluxed for 20 h, and then the solvent was evaporated under reduced pressure. The resulting crude product was taken up in EtOAc (20 mL) and water (20 mL). The separated organic layer was successively washed with 10% aq. K_3_PO_4_ solution (3 x 10 mL) and brine (10 mL), dried over anhydrous Na_2_SO_4_, and evaporated to dryness under reduced pressure. The residue was purified by column chromatography on silica gel (EtOAc/hexanes = 1/9) to afford **6a** (599 mg, 92%) as a yellow solid.

**(*Z*)-3-((Bis(4-iodophenyl)boryl)oxy)-1,3-diphenylprop-2-en-1-one (6a)**

mp 231-232 °C; ^1^H NMR (500 MHz, CDCl_3_) δ_H_ 8.25-8.06 (m, 4H), 7.74-7.65 (m, 2H), 7.64-7.47 (m, 8H), 7.31 (d, *J* = 7.9 Hz, 4H), 7.02 (s, 1H) ppm; ^13^C NMR (125 MHz, CDCl_3_): δ_C_ 183.1, 136.4, 134.8, 133.4, 133.0, 129.2, 128.5, 94.3, 93.1 ppm; ^11^B NMR (160 MHz, CDCl_3_): δ_B_ 9.5 ppm; HRMS-EI *m/z* [M]^+^ calcd for C_27_H_19_O_2_BI_2_, 639.9567, found 639.9563.

**(*Z*)-3-((Bis(3,5-difluorophenyl)boryl)oxy)-1,3-diphenylprop-2-en-1-one (6b)**

410 mg, 89%; yellow solid; mp 275-276 °C; ^1^H NMR (500 MHz, CDCl_3_): δ_H_ 8.21 (d, *J* = 7.5 Hz, 4H), 7.75 (t, *J* = 7.35 Hz, 2H), 7.63 (t, *J* = 7.9 Hz, 4H), 7.08-7.06 (m, 5H), 6.65 (tt, *J* = 2.35, 9.15 Hz, 2H) ppm; ^13^C NMR (125 MHz, CDCl_3_): δ_C_ 183.1, 163.9 (d, *J* = 44.3 Hz), 161.9 (d, *J* = 44.7 Hz), 135.1, 132.6, 129.3, 128.6, 112.9 (dd, *J* = 16.7, 66.95 Hz), 101.9 (t, *J* = 101.35 Hz), 94.4 ppm; ^11^B NMR (160 MHz, CDCl_3_): δ_B_ 8.2 ppm; HRMS-EI *m/z* [M]^+^ calcd for C_27_H_17_O_2_BF_4_, 460.1258, found 460.1247.

**(*Z*)-3-((Bis(4-vinylphenyl)boryl)oxy)-1,3-diphenylprop-2-en-1-one (6c)**

348 mg, 79%; yellow solid; mp 181-182 °C; ^1^H NMR (500 MHz, CDCl_3_): δ_H_ 8.21 (d, *J* = 7.5 Hz, 4H), 7.70 (t, *J* = 7.35 Hz, 2H), 7.61-7.57 (m, 8H), 7.36 (d, *J* = 8.0 Hz, 4H), 7.03 (s, 1H), 6.72 (dd, *J* = 10.9, 17.6 Hz, 2H), 5.71 (d, *J* = 17.7 Hz, 2H), 5.16 (d, *J* = 11.0 Hz, 2H) ppm; ^13^C NMR (125 MHz, CDCl_3_): δ_C_ 183.0, 137.5, 135.9, 134.4, 133.3, 131.6, 129.0, 128.5, 125.3, 112.4, 94.2 ppm; ^11^B NMR (160 MHz, CDCl_3_): δ_B_ 10.3 ppm; HRMS-EI *m/z* [M]^+^ calcd for C_31_H_25_O_2_B, 440.1948, found 440.1943.

**(*Z*)-3-((Di(thiophen-3-yl)boryl)oxy)-1,3-diphenylprop-2-en-1-one (6d)**

312 mg, 78 %; yellow solid; mp 216-217 °C; ^1^H NMR (500 MHz, CDCl_3_): δ_H_ 8.18-8.16 (m, 4H), 7.70-7.67 (m, 2H), 7.57 (t, *J* = 7.8 Hz, 4H), 7.36-7.31 (m, 6H), 7.0 (s, 1H) ppm; ^13^C NMR (125 MHz, CDCl_3_): δ_C_ 182.9, 134.4, 133.2, 131.3, 129.0, 128.5, 126.8, 124.3, 93.4 ppm; ^11^B NMR (160 MHz, CDCl_3_): δ_B_ 8.6 ppm; HRMS-EI *m/z* [M]^+^ calcd for C_23_H_17_O_2_BS_2_, 400.0763, found 400.0769.

**(*Z*)-3-((Diphenylboryl)oxy)-1-(4-methoxyphenyl)-3-(4-(trifluoromethyl)phenyl)prop-2-en-1-one (6e)**

359 mg, 80%; yellow solid; mp 232-233 °C; ^1^H NMR (500 MHz, CDCl_3_) δ_H_ 8.19-8.11 (m, 4H), 7.66-7.58 (m, 4H), 7.31-7.23 (m, 5H), 7.22-7.15 (m, 2H), 7.05-6.96 (m, 4H), 6.83 (s, 1H), 3.91 (s, 6H); ^13^C NMR (125 MHz, CDCl_3_): δ_C_ 183.3, 179.5, 165.5, 136.9, 131.4, 128.4, 127.3, 126.6, 125.9, 125.2, 114.6, 93.9, 55.8 ppm; ^11^B NMR (160 MHz, CDCl_3_): δ_B_ 8.1 ppm; HRMS-EI *m/z* [M]^+^ calcd for C_29_H_25_O_4_B, 448.1846, found 448.1851.

**(1*E*,4*Z*,6*E*)-5-((Diphenylboryl)oxy)-1,7-bis(4-hydroxy-3-methoxyphenyl)hepta-1,4,6-trien-3-one** **(6f)**

331 mg, 62%; red solid; mp 254-259 °C; ^1^H NMR (500 MHz, *d*_6_-DMSO): δ_H_ 10.23 (bs, 2H), 7.98 (d, *J* = 15.5 Hz, 2H), 7.49-7.46 (m, 6H), 7.34 (dd, *J* = 1.6, 8.25 Hz, 2H), 7.19 (t, *J* = 7.1 Hz, 4H), 7.12-7.09 (m, 2H), 6.96 (d, *J* = 15.5 Hz, 2H), 6.87 (d, *J* = 8.1 Hz, 2H), 6.25 (s, 1H), 3.86 (s, 6H) ppm; ^13^C NMR (125 MHz, CDCl_3_): δ_C_ 179.9, 151.1, 148.6, 145.4, 131.5, 127.3, 126.6, 126.3, 124.8, 119.8, 116.3, 112.8, 56.2 ppm; ^11^B NMR (160 MHz, CDCl_3_): δ_B_ 9.1 ppm; HRMS-EI *m/z* [M]^+^ calcd for C_33_H_29_O_6_B, 532.2057, found 532.2064.

**2-Acetyl-3-((diphenylboryl)oxy)-5,5-dimethylcyclohex-2-enone (6g)**

270 mg, 78%; white solid; mp 86-87 °C; ^1^H NMR (500 MHz, CDCl_3_) δ_H_ 7.49-7.38 (m, 4H), 7.34-7.20 (m, 6H), 2.78 (s, 3H), 2.72 (s, 2H), 2.37 (s, 2H), 1.09 (s, 6H) ppm; ^13^C NMR (125 MHz, CDCl_3_): δ_C_ 197.7, 196.1, 193.7, 131.1, 127.5, 127.1, 52.1, 47.3, 30.8, 28.2, 27.3 ppm; ^11^B NMR (160 MHz, CDCl_3_): δ_B_ 10.2 ppm; HRMS-EI *m/z* [M]^+^ calcd for C_22_H_23_O_3_B, 346.1740, found 346.1741.

**VI. Preparation of Four-Coordinate Organoborons from *N,N*-Ligands**

**General Procedure for the preparation of 7a**

To a 100 mL round bottomed flask equipped with magnetic stirring bar and reflux condenser, were added sequentially 2-(5-methyl-1*H*-pyrrol-2-yl)pyridine^8^ (158.2 mg, 1.0 mmol), phenylboronic acid (1.10 g, 9.0 mmol), K_3_PO_4_ (636.8 mg, 3.0 mmol) and 1,4-dioxane (50 mL). The mixture was refluxed for 20 h, and then the solvent was evaporated under reduced pressure. The resulting crude product was taken up in EtOAc (20 mL) and water (20 mL). The separated organic layer was successively washed with 10% aq. K_3_PO_4_ solution (3 x 10 mL) and brine (10 mL), dried over anhydrous Na_2_SO_4_, and evaporated to dryness under reduced pressure. The residue was purified by column chromatography on silica gel (EtOAc/hexanes = 1/9) to afford **7a** (246 mg, 76%) as yellow solid.

**2-(1-(Diphenylboranyl)-5-methyl-1*H*-pyrrol-2-yl)pyridine (7a)**

mp 201-202 °C; ^1^H NMR (500 MHz, CDCl_3_) δ_H_ 8.04 (d, *J* = 6.0 Hz, 1H), 7.72 (t, *J* = 7.6 Hz, 1H), 7.46 (d, *J* = 8.0 Hz, 1H), 7.28-7.21 (m, 10H), 6.88 (t, *J* = 6.0 Hz, 1H), 6.73 (d, *J* = 1.6Hz, 1H), 6.14 (d, *J* = 1.6 Hz, 1H), 2.03 (s, 3H) ppm; ^13^C NMR (125 MHz, CDCl_3_) δ_C_ 149.2, 141.5, 140.3, 137.6, 133.5, 130.4, 127.5, 126.6, 117.7, 115.7, 113.4, 107.2, 13.8 ppm; HRMS-EI *m/z* [M]^+^ calcd for C_22_H_19_BN_2_, 322.1641, found 322.1647.

**1-(Diphenylboryl)-2-(pyridin-2-yl)-1*H*-indole (7b)**

283 g, 79%; yellow solid; mp 255-256 °C (lit.^9^ 255 °C); ^1^H NMR (500 MHz, CDCl_3_): δ_H_ 8.44 (d, *J* = 5.7 Hz, 1H), 8.02-7.99 (m, 1H), 7.74 (d, *J* = 7.8 Hz, 1H), 7.32-7.30 (m, 5H), 7.27-7.24 (m, 7H), 7.15 (s, 1H), 7.12-7.05 (m, 2H) ppm; ^13^C NMR (125 MHz, CDCl_3_): δ_C_ 149.7, 142.5, 140.9, 139.3, 136.5, 133.3, 132.7, 127.6, 126.9, 123.3, 122.2, 121.3, 119.7, 118.7, 114.2, 98.7 ppm; HRMS-EI *m/z* [M]^+^ calcd for C_25_H_19_N_2_B, 358.1641, found 358.1641

**General Procedure for the preparation of 7c**

5-Phenyldipyrromethane (222.3 mg, 1.0 mmol), 1,4-dioxane (50 mL) and DDQ (510.8 mg, 2.25 mmol) were added sequentially to a 100 mL round bottomed flask equipped with magnetic stirring bar and reflux condenser. The mixture was stirred for 30 min at room temperature.^10^ The disappearance of the starting material was confirmed by TLC. To this mixture, were added PhB(OH)_2_ (1.09 mg, 8.9 mmol) and K_3_PO_4_ (656.7 mg, 3.1 mmol) and then the resulting mixtures were heated to reflux for 20 h. Then the solvent was evaporated under reduced pressure and the crude product was taken up in EtOAc (20 mL) and water (20 mL). The separated organic layer was successively washed with 10% aq. K_3_PO_4_ solution (3 x 10 mL) and brine (10 mL), dried over anhydrous Na_2_SO_4_, and evaporated to dryness under reduced pressure. The residue was purified by column chromatography on silica gel (EtOAc/hexanes = 3/7) to afford **7c** (292 mg, 76%) as an orange solid.

**(*Z*)-1-(Diphenylboranyl)-2-(phenyl(2*H*-pyrrol-2-ylidene)methyl)-1*H*-pyrrole (7c)**

mp 185-186 °C; ^1^H NMR (500 MHz, CDCl_3_): δ_H_ 7.65-7.59 (m, 4H), 7.59-7.54 (m, 1H), 7.54-7.49 (m, 2H), 7.29-7.23 (m, 5H), 7.23-7.17 (m, 6H), 6.97 (dd, *J* = 4.2, 1.3 Hz, 2H), 6.52 (dd, *J* = 4.3, 1.8 Hz, 2H) ppm; ^13^C NMR (126 MHz, CDCl_3_) δ_C_ 146.8, 145.0, 134.7, 134.6, 132.8, 130.5, 130.2, 129.1, 128.19, 127.4, 126.2, 117.5 ppm; ^11^B NMR (160 MHz, CDCl_3_): δ_B_ 1.4 ppm; HRMS-EI *m/z* [M]^+^ calcd for C_27_H_21_N_2_B, 384.1798, found 384.1791.

**(*Z*)-1-(Bis(4-(trifluoromethyl)phenyl)boranyl)-2-(phenyl(2*H*-pyrrol-2-ylidene)methyl)-1*H*-pyrrole (7d)**

375 mg, 72%; orange solid; mp 179-180 °C; ^1^H NMR (500 MHz, CDCl_3_) δ_H_ 7.74-7.67 (m, 3H), 7.54 (ddt, *J* = 8.7, 6.8, 1.6 Hz, 2H), 7.51-7.44 (m, 6H), 7.20 (t, *J* = 1.5 Hz, 3H), 6.67 (dd, *J* = 4.2, 1.3 Hz, 3H), 6.23 (dd, *J* = 4.2, 1.8 Hz, 3H) ppm; ^13^C NMR (126 MHz, CDCl_3_) δ_C_ 145.80, 144.97, 134.49, 134.30, 131.48, 130.69, 130.09, 128.72, 128.18, 128.04, 127.93, 123.83, 123.80, 117.93 ppm; ^11^B NMR (160 MHz, CDCl_3_): δ_B_ 1.8 ppm; HRMS-EI *m/z* [M]^+^ calcd for C_29_H_19_N_2_F_6_B, 520.1545, found 520.1539.

**VII. References**

1.Wu, Q., Esteghamatian, M., Hu, N.-X., Popovic, Z., Enright, G. Tao, Y.,D’lorio, M. & Wang, S. Synthesis, structure, and electroluminescence of BR_2_q (R = Et, Ph, 2-Naphthyl and q = 8-hydroxyquinolato). *Chem. Mater.* **12**, 79-83 (2000).

2. Benkovic S. J. *et al*. Identification of borinic esters as Inhibitors of bacterial cell growth and bacterial methyltransferases, CcrM and MenH. *J. Med. Chem.* **48**, 7468-7476 (2005).

3. Cui, Y., Liu, Q.-D., Bai, D.-R., Jia, W.-L., Tao, Y. & Wang, S. Organoboron compunds with an 8-hydroxyquinolato chelate and its derivatives: substituent effects on structures and luminescence. *Inorg. Chem.* **44**, 601-609 (2005).

4. Farfan, N., Castillo, D., Joseph-Nathan, P., Contreras, R, & Szentplay, L. V. Through-bond modulation of N-B ring formation shown by NMR and X-ray diffraction studies of borate derivatives of pyridyl alcohols. *J. Chem. Soc., Perkin Trans. 2,* 527-532 (1992).

5. Kim, N. M., Shin, C. H., Lee, M. H. & Do, Y. Four-coordinate boron compounds from 2-(2-pyridyl) phenol ligand as novel hole-blocking materials for phosphorescent OLEDs. *J. Organomet. Chem*. **694**, 1922-1928 (2009).

6. Cade, I. A. & Ingelson, M. J. *syn*-1,2-Carboboration of alkynes with borenium cations. *Chem. Eur. J.* **20**, 12874-12880 (2014).

7. Berionni, G., Leonov, A. I., Mayer, P., Ofial, A.R. & Mayr, H. Fine-tuning the nucleophilic reactivities of boron ate complexes derived from aryl and heteroaryl boronic esters. *Angew. Chem. Int. Ed.* **54**, 2780-2783 (2015).

8. Chen, H.-Y. *et al*. Rational color tuning and luminescent properties of functionalized boron-containg 2-pyridyl pyrrolide complexes. *Adv. Funct. Mater.* **15**, 567-574 (2005).

9. Liu, S.-F. *et al*. Syntheses, structures, and electroluminescence of new blue/green luminescent chelate compounds: Zn(2-py-in)_2_(THF), BPh_2_(2-py-in), Be(2-py-in)_2_, and BPh_2_(2-py-aza) [2-py-in = 2-(2-pyridyl)indole; 2-py-aza = 2-(2-pyridyl)-7-azaindole]. *J. Am. Chem. Soc.* **122***,* 3671-3678 (2000).

10. Wagner, R. W. & Lindsay, J. S. Boron-dipyrromethene dyes for incorporation in synthetic multi-pigment light-harvesting arrays. *Pure Appl. Chem.* **68**, 1373-1380 (1996).

**Copies of ^1^H & ^13^C NMR spectra for all compounds**


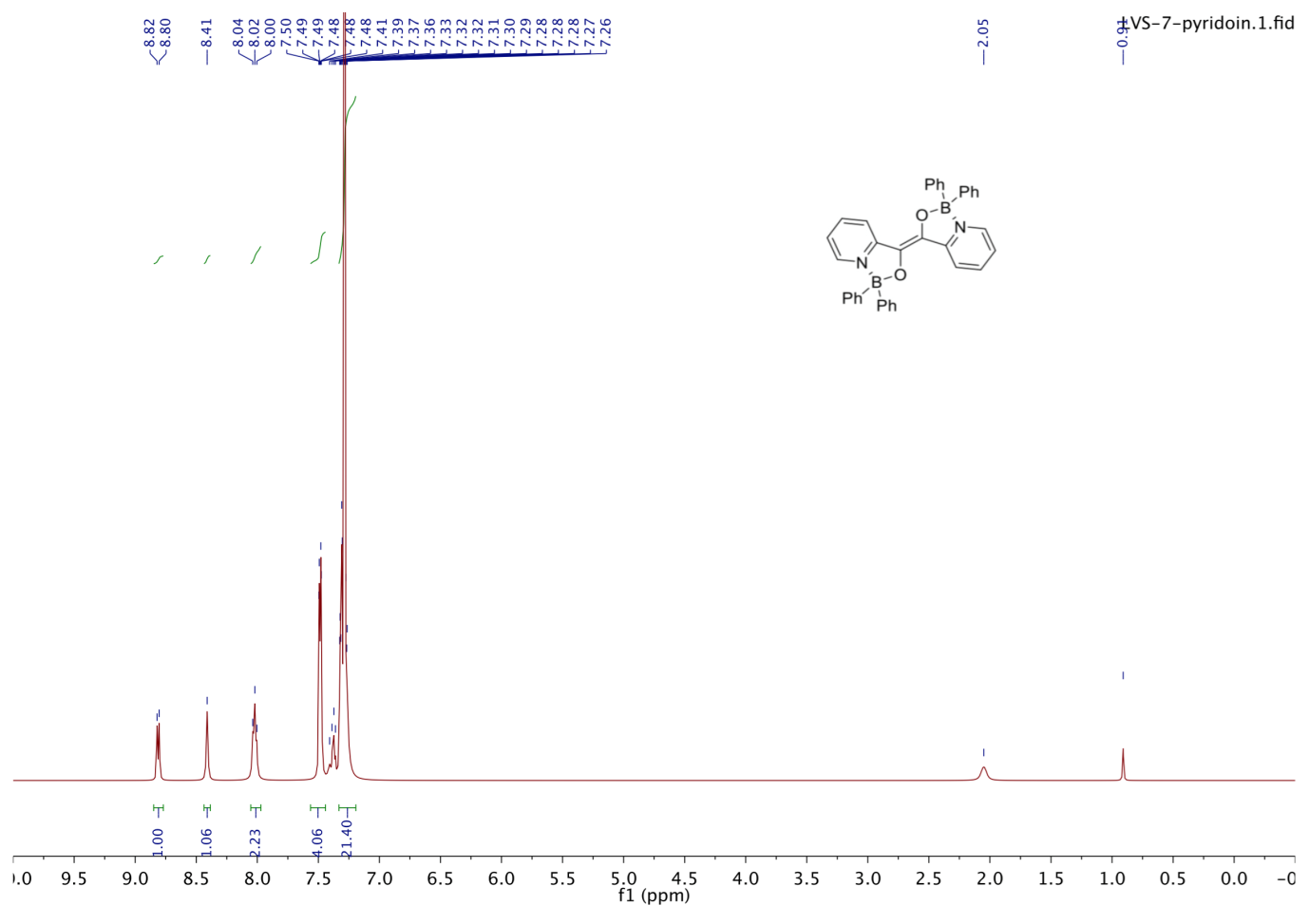


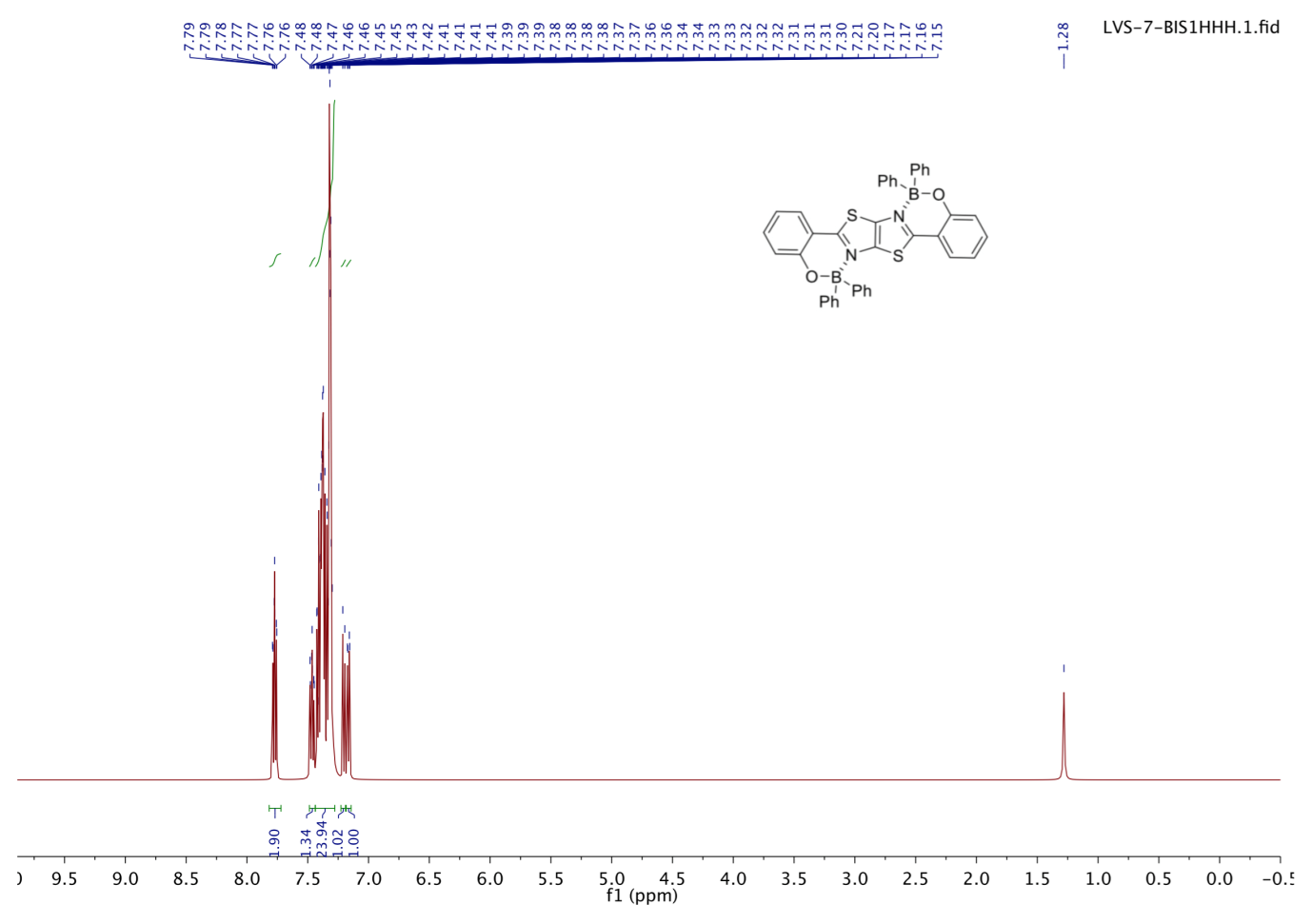


**Copies of ^11^B NMR spectra for selected compounds**
